# Time-Resolved Circular Dichroism in Molecules: Experimental and Theoretical Advances

**DOI:** 10.3390/molecules29174049

**Published:** 2024-08-27

**Authors:** Marta Monti, Leonardo Biancorosso, Emanuele Coccia

**Affiliations:** 1 The Abdus Salam International Centre for Theoretical Physics, Strada Costiera 11, 34151 Trieste, Italy; 2 Dipartimento di Scienze Chimiche e Farmaceutiche, University of Trieste, Via L. Giorgieri 1, 34127 Trieste, Italy; leonardo.biancorosso@phd.units.it

**Keywords:** molecular dynamics, electronic excited-state properties, enantiomers

## Abstract

Following changes in chirality can give access to relevant information on the function or reactivity of molecular systems. Time-resolved circular dichroism (TRCD) spectroscopy proves to be a valid tool to achieve this goal. Depending on the class of molecules, different temporal ranges, spanning from seconds to femtoseconds, need to be investigated to observe such chiroptical changes. Therefore, over the years, several approaches have been adopted to cover the timescale of interest, especially based on pump–probe schemes. Moreover, various theoretical approaches have been proposed to simulate and explain TRCD spectra, including linear and non-linear response methods as well as non-adiabatic molecular dynamics. In this review, an overview on both experimental and theoretical advances in the TRCD field is provided, together with selected applications. A discussion on future theoretical developments for TRCD is also given.

## 1. Introduction

**Figure 1:**
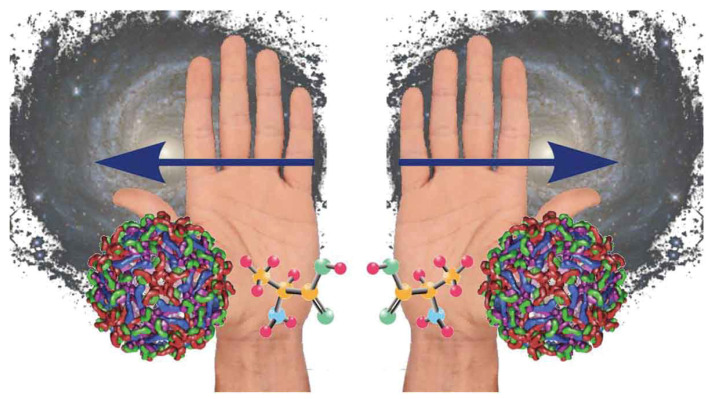
Examples of different chiral systems that exist, from molecules to astronomical objects. Reproduced from Ref. [1].

Chirality is the geometrical property of an object which cannot be superimposed with its mirror image by any translation or rotation. As shown in Figure 1, a great variety of systems displays this property, from elementary particles to astronomical objects, including our hands, which actually gave origin to the name itself (*keir* in Greek means hand) [1]. In chemistry, the two non-superimposable images are referred to as enantiomers. Chiral molecules show the exact same chemical and physical properties but act differently in a chiral environment and have opposite optical rotation. Indeed, when interacting with polarized radiation, one enantiomer will rotate the plane clockwise (dextrorotation, +enantiomer), while the other will rotate the plane anticlockwise (levorotation, −enantiomer). This unique optical activity has made chiral materials fascinating for many applications in biology, chemistry, and physics. For instance, chirality is exploited for drug design and catalysis applications, as well as for enantioselective synthesis and chiral sensing [2,3,4,5,6]. Chirality also plays a major role in life as we know it, since several classes of biomolecules, such as amino acids, nucleic acids, and sugars, present this property. Remarkably, only one of the two series of enantiomers can be found in nature for these chiral biomolecules (i.e., only l-amino acids and d-sugars). The key question of why the evolution of living organisms has led to homochirality is still unanswered, but many hypotheses have been proposed [7,8,9,10].

Focusing now on their peculiar optical activity, it is worth mentioning that different types of chiral matter–light interactions can be investigated, such as electronic absorption, vibrational absorption, or luminescence. Corresponding spectroscopic techniques have been developed to characterize the optical properties of chiral systems, namely electronic circular dichroism (ECD) and optical rotatory dispersion (ORD) [11], vibrational circular dichroism (VCD) [12], or circularly polarized luminescence (CPL) [13]. Depending on the selected technique, conformational and structural changes in different electronic states can be investigated. In this review, our attention will be focused on ECD (or simply CD), and in particular on its ultrafast time-resolved description, with a brief mention of the ORD technique.

CD is defined as the differential absorption of left- and right-handed circularly polarized light, $A_{L}$ and $A_{R}$, respectively, by a chiral molecule:

(1) $$CD=A_{L}-A_{R}.$$

From Equation (1), it is clear that CD signals are non-negligible, and thus can be measured only in correspondence to absorption bands. Similarly to the Lambert–Beer law, one can define a molar quantity (Δϵ), that is, the difference in the molar extinction coefficients, as:

(2) $$\Delta \epsilon =\frac{CD}{l\times c}$$

where *l* and *c* are the optical path length and the concentration of the sample, respectively. Chiral systems also possess the ability to rotate the polarization plane of a linearly polarized radiation, i.e., ORD. Over the years, CD spectroscopy has become much more popular and used compared to ORD, even though they yield identical information due to their Kramers–Kronig relation [14]. One of the main advantages of this spectroscopy is its high sensitivity to the absolute configuration and conformational changes, which can often not be detected in standard absorption spectra [15]. This characteristic has made CD spectroscopy a widely employed technique to probe the secondary structure of biomolecules as peptides, proteins, and DNA. Indeed, different conformations, such as α-helix, β-sheet, or random coil, present specific CD peaks, usually in the range of 190–250 nm. Moreover, energy position and intensity of CD peaks can change as a function of the adopted temperature, pH, and solvent [16]. Therefore, a CD spectrum provides much relevant information regarding the structure of the sample and its relation with the environment.

Magnetic circular cichroism (MCD) spectroscopy is instead based on the concept of magnetic optical activity (MOA) [17,18], i.e., optical effects occurring when electromagnetic radiation interacts with matter in the presence of a magnetic field that is aligned with the direction of light propagation. Magnetic Optical Rotation (MOR) and MCD are the most important MOA effects [18]. MCD arises from the difference in absorption coefficients when the sample is irradiated with left- and right-circularly polarized light, while MOR results from the difference in the refractive indices of the sample under the same conditions. MCD spectroscopy is widely used to explore the geometric, electronic, and magnetic properties of chemical systems. This technique is applicable to systems in the gas phase, in solution, or to solids, making it a versatile tool in chemical research [17,18,19,20].

**Figure 2:**
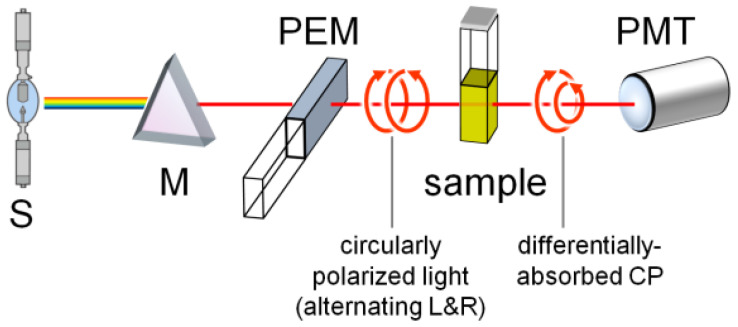
Schematic representation of a spectrophotometer for steady-state CD measurements. S: source, M: monochromator, PEM: photo-elastic modulator, PMT: photomultiplier detector. Reproduced from Pescitelli, G. Electronic Circular Dichroism. Encyclopedia. Available online: https://encyclopedia.pub/entry/67 (accessed on 7 July 2024). License: https://creativecommons.org/licenses/by-sa/4.0/.

The most common way of measuring steady-state CD spectra consists in the direct detection of the different absorbance of left- and right-circularly polarized light. As shown in Figure 2, first a source (S) of radiation passes through a monochromator (M). The resulting linearly polarized light then becomes circular using a photo-elastic modulator (PEM). Left- and right-circularly polarized radiations are produced alternately by the PEM with a frequency of ca. 50 kHZ. The difference between the two transmitted signals corresponds to the CD signal, which is first amplified by means of a lock-in amplifier and then collected by a photomultiplier detector (PMT in Figure 2). Alternatively, one could measure the ellipticity (θ) that a chiral sample induces on a linearly polarized source, which can be considered as the result of two equal superimposed circularly polarized waves. When the linearly polarized light passes through a chiral sample, the two waves are absorbed differently and the transmitted radiation becomes elliptical.

To capture the temporal changes in a chiral signal, switching from static to time-resolved CD (TRCD) spectroscopy is mandatory. Depending on the process under investigation, the timescale of interest in TRCD measurements can change significantly, spanning from seconds to femtoseconds. For instance, the conformational changes in macromolecules can be measured with a time resolution of milli-/microseconds [21]. Instead, a time resolution in the order of nanoseconds allows following the changes in the secondary structure of proteins during the folding [22]. Going down to the picosecond time resolution, it is possible to investigate excited-state dynamics and fluorescence. Finally, working with a femtosecond resolution, which is the timescale of molecular vibrations, processes such as formation and breaking of chemical bonds, isomerization, and the stereochemistry of photo-excited systems can be properly studied. Moreover, while global changes in the secondary structure of proteins and DNA occur on longer timescales, as mentioned above, local changes involving small portions of the system occur on a femtosecond timescale.

The most commonly used setup for TRCD is a pump–probe scheme In a pump–probe experiment, the sample is excited by a first pulse (the pump), and then its response is measured through a second pulse (the probe) after a certain time delay. Specific details of TRCD spectroscopy within a pump–probe setup are given in Section 2. Although investigating the chirality of excited systems is extremely challenging, a great effort has been made over recent decades to develop suitable ultrafast TRCD setups due to the incredible potential of this technique.

Here, we give a detailed overview of experimental and theoretical approaches to study TRCD in molecular systems. Experimental TRCD advances are discussed in Section 2, while theoretical methods are explained in Section 3. The review is concluded discussing the possible use of time-domain quantum methodologies for TRCD, providing an original viewpoint on future perspectives in Section 4. A detailed overview of experimental and theoretical advances in time-resolved MCD is beyond the scope of this work. A few details about X-ray MCD (XMCD) [23,24] and its time-resolved formulation [25,26,27] are given in Section 2.4.

## 2. TRCD: Experimental Aspects

Over the years, several experimental setups have been implemented for measuring TRCD spectra. Starting from the 1970s, the first attempts were made by combining the standard CD detection with stopped flow [21,28], temperature-jump [29], and flash photolysis [30] methods to induce and study conformational changes in biomolecules, especially over the micro-/millisecond time regime. Many applications are available, with a particular focus on the protein/DNA folding problem [21,31,32,33,34,35] and the study of photo-intermediates [36,37,38,39,40]. However, the first two techniques have been experimentally limited by their poor time resolution for a long time, while the latter has been limited by the presence of photoinduced artifacts. Several reviews are available for a much wider description of TRCD experiments in this timescale [41,42,43,44].

In order to move to shorter timescales, i.e., the sub-picosecond and femtosecond time-domain, a pump–probe setup needs to be implemented [45,46]. The idea is to first electronically excite the sample by using a short laser pulse and then to follow the CD changes induced by this pump by means of a second delayed pulse, known as the probe pulse, which can be either circularly or linearly polarized (see Section 2.1). The great advantage is that the time resolution of a pump–probe experiment only depends on the duration of the two pulses. However, despite the simplicity of the principle behind the technique, it is actually quite challenging to implement this setup for TRCD detection. First, TRCD signals are significantly weaker (i.e., two to five orders of magnitude) than those measured in standard transient absorption experiments, thus making it hard to distinguish them from the noise. Furthermore, the pump–probe geometry can induce polarization artifacts, which are difficult to remove [44]. Over recent years, a lot of effort has been devoted to overcoming these drawbacks due to the possibility of studying chirality changes on a short or ultrashort timescale with pump–probe TRCD.

In this Section, we focus on fast and ultrafast TRCD. The history and most relevant experimental developments in the ultrarapid TRCD field are discussed in Section 2.1, Section 2.2 and Section 2.3, while CD coupled to high-harmonic generation and photoelectron spectroscopy is shown in Section 2.4.

### 2.1. Ultrafast TRCD: Differential Absorption and Ellipsometric Setup

**Figure 3:**
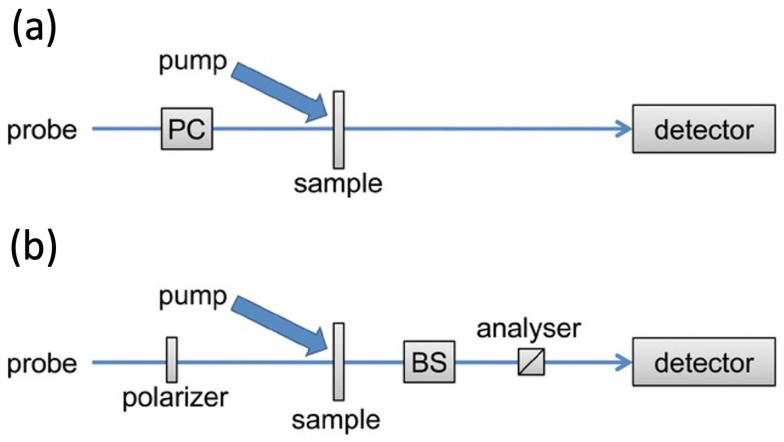
Schematic representation of the pump–probe setups employed for measuring ultrafast TRCD. Panel (**a**) shows the differential absorption setup where a circularly polarized probe pulse is used. The probe polarization is modulated through a Pockels cell (PC). Panel (**b**) shows the ellipsometric setup where a linearly polarized probe pulse becomes elliptical after interacting with the sample. The change in polarization is measured combining a Babinet–Solei (BS) compensator and a cross-polarized analyzer. Adapted with permission from Ref. [45]. Copyright © 2013 Wiley-Liss, Inc.

Two main approaches are widely used for ultrafast TRCD measuraments: differential absorption TRCD and ellipsometric TRCD, which are schematized in Figure 3 [45]. The former scheme resembles that of a steady-state CD experiment; hence, the difference in the absorption of left- and right-circularly polarized light is measured with a circularly polarized probe. The latter approach exploits the fact that a linearly polarized probe pulse becomes elliptical when passing through a chiral sample. Therefore, setups that can measure pump-induced changes in ellipticity have been developed over the years. Differential absorption TRCD measurements are more straightforward but limited by the strong absorption of the achiral background, while an ellipsometric TRCD setup can provide background-free detection, improving the sensitivity of the response. It is also worth highlighting that this latter setup provides the possibility of measuring both CD and ORD temporal changes.

**Figure 4:**
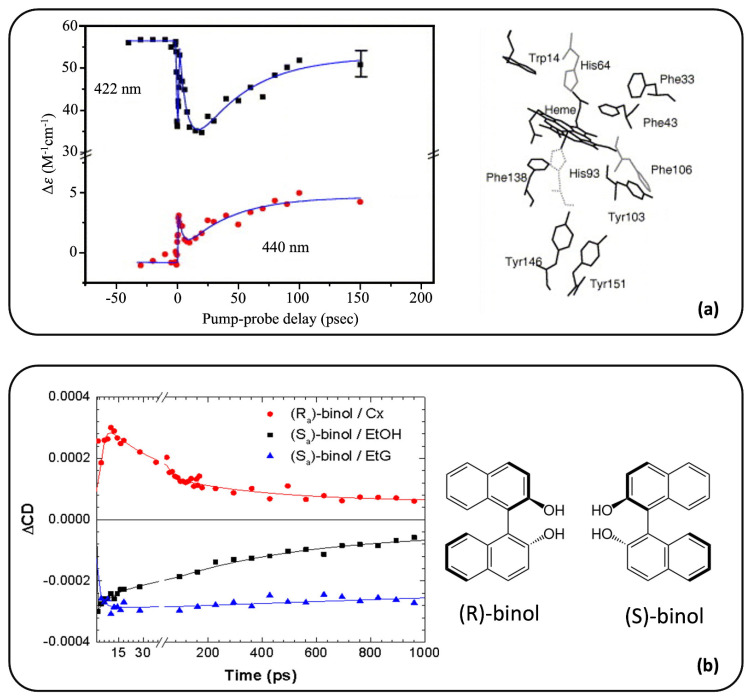
Applications of TRCD spectroscopy in the sub-picosecond time-domain and single-wavelength detection mode. Panel (**a**) shows the CD behavior as a function of the pump–probe delay at 422 nm (black squares) and 440 nm (red dots), which are close to the MbCO and Mb peaks, respectively. A sketch of the Mb residues involved in the CD signals is also reported in the same panel. Adapted with permission from Ref. [49]. Copyright © 2005 Elsevier B. V. Panel (**b**) shows normalized TRCD changes in the ps scale at 234 nm for (R)−binol in cyclohexane (Cx, red dots) and (S)-binol in both ethanol (EtOH, black squares) and ethylene glycol (EtG, blue triangles). A schematic representation of the two binol enantiomer structures is also reported in the same panel. Adapted with permission from Ref. [55]. Copyright © 2019 American Chemical Society.

First measurements with a time resolution of (few) nanoseconds were realized by Lewis and collaborators in 1985 [47]. Adopting an ellipsometric TRCD scheme, they were able to follow the CD changes occurring in carbonmonoxy-myoglobin (MbCO) upon photolysis. In 1989, Xie and Simon [48] were the first to perform experiments in the picosecond domain, modulating the TRCD detection with a Pockels cell. The first sub-picosecond TRCD measurement was performed in 2005 by Dartigalongue and Hache [49], providing new results on the MbCO conformational changes that occur in photolysis. Here, the MbCO molecules are first pumped at 400 nm and then probed with a pulse working between 415 and 450 nm. Panel (a) of Figure 4 shows the CD evolution as a function of the pump–probe delay for two specific wavelengths, i.e., λ = 422 nm and λ = 440 nm. The two curves show similar temporal behaviors, which are defined by a decrease around 7 ps and an increase around 43 ps. After 100 ps, no other changes are visible in the CD signals. Therefore, they observe a transient change in the CD within 10 ps and then a relaxation towards the steady-state CD of Mb in the sub-100 ps range. In this paper, the authors assign the sub-10 ps feature to a transient conformation where the proximal histidine moves due to its compression between the domed (i.e., non-planar) heme and the F-helix. The local stress the histidine is suffering is gradually released while the F-helix reaches its steady-state.

In 2006, Niezborala and Hache [50] proposed a new technique based on measuring the change in the probe ellipticity induced by the pump by combining a Babinet–Soleil (BS) compensator with a crossed analyzer. The great advantages of this ellipsometric TRCD setup are that no probe modulation is required, and the measurements are free of most artifacts, even though they are sequential, and thus very time-consuming. The setup was tested on a chiral complex of ruthenium and applied to the photolysis of MbCO, showing its ability to detect even weak pump-induced CD changes. Notably, the approach proposed here also allows performing ORD measurements by rotating the analyzer instead of the BS compensator. ORD changes in the MbCO photolysis are shown in the original work [50]. Hache and collaborators have shown in several other works that ultrafast ellipsometric TRCD in single-wavelength detection mode is well suited for studying the CD changes in proteins and other chiral compounds [51,52,53,54,55].

Panel (b) of Figure 4 shows the ultrafast TRCD spectra recently measured for (R)- and (S)-binol in different solvents (i.e., cyclohexane, ethanol, and ethylene glycol) [55]. The two enantiomers were electronically excited at 266 nm and their chiroptical changes were followed using a probe at 234 nm, which corresponds to the region where the largest intensity of the static CD band is observed. Interestingly, sign inversion of the CD signals can be noticed comparing the steady-state and the excited response after 1 ps at the probe wavelength. Indeed, the CD peak of (S)-binol in ethanol is positive in the ground-state, while its depletion, and thus the excitation, leads to a negative peak. An opposite trend is found instead for (R)-binol in cyclohexane: negative signal in the ground-state, positive peak in the excited state. Therefore, significant changes in the CD response can be observed due to variations in the electronic structure and conformation of the sample upon excitation.

All the mentioned TRCD measurements were performed with single-wavelength detection, providing information, for instance, on the MbCO photolysis [48,56] or the ring opening mechanism of provitamin D [57]. It is worth noting that the latter system has also been investigated from a theoretical perspective [58], and the adopted computational approach and results are discussed in Section 3. However, gaining the global spectral response would provide much more insights, considering that CD signals are wavelength-dependent. In order to accomplish that with these schemes, one would need to accumulate a significant number of measurements with different probe wavelengths, which is, of course, extremely time-consuming. Therefore, this limitation has pushed researchers towards the development of broadband spectral detectors, which are discussed in the next section.

### 2.2. Broadband TRCD Detection

In 2010, Trifonov et al. [59] performed the first broadband TRCD experiment, with a differential absorption setup, probing simultaneously the whole visible range. In this work, the CD signals of bis-merocyanine nanorod aggregates in a solution of tetrahydrofurane and methylcyclohexane were measured by using a white-light continuum probe. The modulation of the probe circular polarization was realized combining a quarter-waveplate and a Pockels cell. Adopting this scheme, good quality of the circular polarization of the probe was achieved all over the whole investigated spectral window, while the accuracy of the TRCD signals (about 1 milli optical density (mOD)) was limited by the use of a charge-coupled device (CCD) camera for the detection.

**Figure 5:**
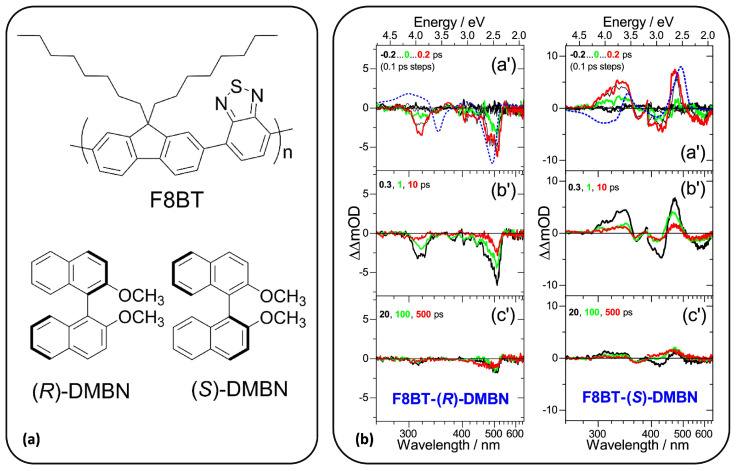
Ultrafast UV-vis broadband TRCD application for a cholesteric thin film of F8BT-(R)-DMBN and F8BT-(S)-DMBN. A sketch of the chemical structure of the achiral F8BT copolymer and the helicene-like DMBN chiral additive is shown in panel (**a**). The TRCD spectra are measured for both the enantiomers at increasing time delays, from −0.2 to 500 ps, as shown in subgraphs a’, b’, and c’ of panel (**b**). Black, green, and red lines represent the transient CD spectra recorded at specific time delays (e.g., −0.2, 0, 0.2). Steady-state CD spectra are also reported for both enantiomers as dotted blue lines in subfigures a’. Adapted with permission from Ref. [61]. Copyright © 2020 American Chemical Society.

In 2019, a similar scheme was presented by Scholz and collaborators [60], who employed a faster readout, reaching an accuracy around 0.3 mOD. In more detail, they implemented a TRCD setup which covers a wide spectral range, from 260 to 700 nm, using a multi-filament supercontinuum, whose fluctuations were recorded shot-to-shot in order to increase the signal-to-noise ratio. This TRCD scheme allowed for probing the dynamics of a polyfluorene–phenylene copolymer in cholesteric thin films. In 2020, the same group re-adopted this scheme to continue investigating chiral cholesteric copolymer films [61]. Here, they studied the strong CD response of an achiral polyfluorene–benzothiadiazole (F8BT) copolymer mixed with chiral binaphthalene (DMBN) derivatives. A scheme of the F8BT-(R)-DMBN and F8BT-(S)-DMBN chemical structures is reported in panel (a) of Figure 5. The ultrafast broadband TRCD spectra measured at different time delays are also shown in Figure 5 (panel (b)). For both enantiomers, the highest energy feature around 490 nm resembles that of the corresponding steady-state CD (blue dotted line), while significant differences are detected in the UV energy range. Indeed, the CD bands located around 315 nm display a sign inversion on a sub-picosecond timescale (i.e., up to 0.2 ps) when the system is in its excited state. In that work, the authors suggest that the UV feature in the TRCD spectra (excited-state sample) arises from S_1_ → S_*n*_ transitions, while it is known that the corresponding band in the steady-state CD spectra (ground-state sample) originates from the S_0_ → S_9_ transition, according to simulations, which has a $\pi \pi^{*}$ character [62]. The probe wavelength ranges adopted in the work allow for a sampling of different upper S_*n*_ excited states, and thus for different transitions, which can lead to significant changes in the CD response. Moreover, they showed that a charge-pair state is formed over this timescale, although with a 30% yield, which will also contribute to the differences in the CD spectra. As shown in panel (b) of Figure 5, the TRCD spectral evolution is followed up to 500 ps, where a relevant reduction in CD intensities is observed due to the depletion of the excited-state population.

In 2022, an improvement of the same TRCD scheme was presented [63], switching from a β barium borate Pockels cell to a potassium dideuterium phosphate crystal Pockels cell for the modulation of the probe polarization. This refined setup provides a better time resolution (100 fs), an increased intensity of the multifilament supercontinuum, as well as an improved signal-to-noise ratio because of its reduced intensity fluctuations. Another improved version of this TRCD setup was presented in 2024 [64] using randomly polarized ultra-short light pulses to minimize the well-known pump-induced artifacts. This technique, defined as TRCD with pump modulation, was tested again on the F8BT copolymer with a helicene-like chiral additive, providing almost perfect mirror images TRCD spectra for thin films with opposite chirality.

In this context of TRCD studies of chiral aggregates, it is also worth mentioning recent work by Ress et al. [65], who focused on the excitonic dynamics of a squaraine polymer in acetone more than on its conformational dynamics. Moreover, they also proposed a different TRCD setup, which does not require elements to modulate the polarization of the probe. Indeed, the key element here is the generation of circularly polarized pulses through a polarization grating. By means of a specific chopping scheme, it is possible to switch between the two pulses and measure TRCD spectra with shot-to-shot detection in the femtosecond regime. Despite all these remarkable progresses, one of the main limitations regards the accuracy of broadband TRCD detection, which, for this reason, has been mostly employed with systems displaying very large TRCD signals.

**Figure 6:**
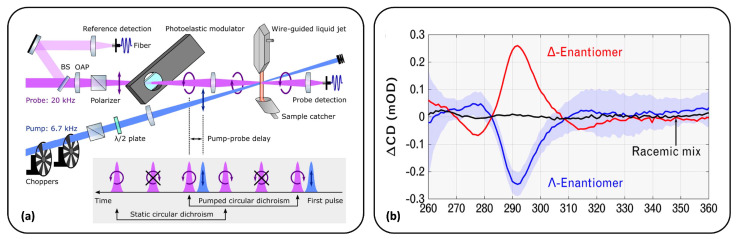
Ultrafast UV-vis broadband TRCD application for Δ and Λ enantiomers of [Ru(bpy)_3_]^2+^. A schematic representation of the setup adopted in this work is reported in panel (**a**). TRCD spectra of both enantiomers and their racemic mixture are shown in panel (**b**). The spectra were measured between 260 nm and 360 nm at 50 ps time delay. Adapted with permission from Ref. [66]. Copyright © The Optical Society.

Recently, Oppermann et al. [66] have proposed a new strategy to significantly improve the accuracy of detection in a broadband fashion. In more detail, they used a TRCD spectrometer with femtosecond time resolution (500 fs) and broadband detection in the deep-UV range (250–370 nm). In this case, the shot-to-shot polarization switching was achieved using a PEM as an alternative to the Pockels cell adopted in previous examples. For the probe detection instead, a double-array metal-oxide-semiconductor detector was employed. A schematic representation of the setup is reported in Figure 6 (panel (a)). The dispersive detection of the probe is preceded by the scrambling of its polarization, which minimizes the setup sensitivity to artifacts. As a result, Oppermann and co-workers were able to successfully record both static and transient CD spectra with an unprecedented sensitivity of <1 mdeg (≈10 μOD). In this first work, the measurements were performed on an enantiopure complex of ruthenium $\left({\left[{\mathrm{Ru}(\mathrm{bpy})}_{3}\right]}^{2+}\right)$ (see Figure 6, panel (b)), also previously investigated by Kliger and co-workers with their TRCD setup with nanosecond time resolution in the 1980s [67,68].

Covering the deep-UV energy range in broadband TRCD detection allows probing the dynamics of biological compounds as nucleobases and aromatic amino acids. Going below 250 nm, it would be possible to access the dynamics of the backbone of polypeptides and proteins, which defines the conformation of the secondary structure. Unfortunately, the generation of proper broad UV probes in this lower energy range is still an unresolved challenge. Oppermann and collaborators overcame this technical limitation [69], substituting backbone carbonyl oxygen atoms with sulfur, massively red shifting (more than 60 nm) the lowest $\pi \pi^{*}$ and n$\pi^{*}$ transitions of the peptide bonds in the energy window accessible by broadband detection. The previously proposed setup combined with this site-specific substitution has proven to be a valid tool to probe even small conformational changes in the peptide backbone in the picosecond time-domain.

Fewer examples are available for broadband ellipsometric TRCD detection with femtosecond time resolution. In this context, it is worth mentioning the work of Mangot and co-workers, who proposed an original setup in 2010 [70]. They combine a pump–probe scheme with a transmission ellipsometer which can measure ORD and also circular dichroism when a broadband quarter-waveplate is added. As in previous works [59], a CCD camera was employed for recording the spectral intensities. The approach presented here was tested on the Δ and Λ enantiomers of the $\left({\left[{\mathrm{Ru}(\mathrm{bpy})}_{3}\right]}^{2+}\right)$ complex.

### 2.3. Single-Shot Ellipsometric TRCD

Differential absorption and ellipsometric TRCD measurements require modulation of the probe circular polarization and a variable phase delay on the probe linear polarization, respectively. Since these procedures are slow and sensitive to the fluctuations of the pump and probe pulses, which influence the signal-to-noise ratio, alternative detection methods have been proposed over the years, such as single-shot measurements with heterodyne interferometry and the balanced method. In 2012, Eom et al. [71] adopted heterodyne spectral interferometry to extract the chiral information directly from the phase and amplitude of the electric field transmitted by a chiral sample after the interaction with linearly polarized radiation. This signal field is known as the optical activity free induction decay field (OA FID). The interaction between a chiral sample and a linearly polarized pulse generates both parallel and perpendicular signal fields, with the latter carrying the information on the chirality of the molecule. The ratio between the two signal fields can be directly related to CD and ORD spectra. Each signal field can be measured with the heterodyne detector using the same local oscillator (LO) beam with a fixed delay. This way, the spectral interferograms, both parallel and perpendicular, are obtained and transformed with the Fourier transform spectral interferometry procedure. The two field signals are measured simultaneously by combining a polarizing beam splitter and a CCD camera. As a result, power and phase fluctuations are both eliminated, leading to a significant improvement of the signal-to-noise ratio. The feasibility of realizing CD and ORD experiments in the near-/mid-IR range with this setup was demonstrated using Ni-based chiral complexes as a test case. In 2015, Hiramatsu and Nagata [72] extended broadband TRCD spectroscopy with a femtosecond time resolution and heterodyne detection to the visible range. Their setup was successfully tested on the well-known $\left({\left[{\mathrm{Ru}(\mathrm{bpy})}_{3}\right]}^{2+}\right)$ complex in aqueous solution. These measurements are extremely susceptible to artifacts induced by the pump pulse, i.e., linear birefringence and linear dichroism. However, they were able to improve the sensitivity of the technique (<0.4 mdeg), thus extracting only the true TRCD signals, with the aid of singular value decomposition analysis. While a great advantage of heterodyne detection is its ability to amplify weak chiral signals by means of the LO beam, a critical drawback is the phase stability of the interferometer. A common-path interferometer has been recently adopted because of its high phase stability, hence enabling broadband ORD and CD measurements in the visible/near-IR range [73,74,75].

More recently, Changenet and Hache [76] developed an ellipsometric setup with a balanced detection geometry. Precisely, a fully symmetrical balanced detection configuration is adopted to avoid the well-known pump-induced artifacts affecting these measurements. In more detail, the two circularly polarized components of the transmitted probe are characterized by combining a quarter-waveplate and a Wollaston prism. The great advantage of adopting this combination is the possibility of detecting CD signals with a single laser shot, thus drastically reducing the acquisition time. The setup proposed here, whose detailed description can be found in Refs. [46,76], was tested on the Δ and Λ enantiomers of $\left[\mathrm{Ru}{\left(\mathrm{phen}\right)}_{3}\right]\cdot 2PF_{6}$ in acetonitrile with an accuracy of 1 mdeg (0.1 mOD) and sub-picosecond time resolution. This chiral complex displays strong CD signals in the 230–330 nm energy range due to the propeller-twist arrangement of the ligands. Interestingly, the excitation of the lowest singlet excited state, which corresponds to a metal-to-ligand charge transfer (MLCT) state, leads to an intersystem crossing on a very short (<100 fs) timescale, and hence to the formation of a long-lived triplet state (^1^$\mathrm{MLCT}\to^{3}\mathrm{MLCT}$). The TRCD changes measured in this work are consistent with the reduction in the excitonic coupling between the ligands induced by the excitation in the UV-visible range [77].

### 2.4. High-Harmonic Generation and Photoelectron Measurements for CD Applications

We provide here a quick overview of the experimental techniques that allow investigating chirality on the femtosecond scale, emphasizing time-resolved setups [78], i.e., photoelectron spectroscopy (PE) [79] and high-harmonic generation (HHG) spectroscopy applied to CD [80,81,82]. Such methodologies have been successfully applied to probe chemical reactions [83,84], to investigate CD in excited electronic states [85] and chiral vibrational dynamics [86], or to follow changes in chirality along photoinduced relaxation in molecules [87,88,89]. Time-resolved PE circular dichroism (TR-PECD) and HHG allow one to investigate shorter timescales, thus overcoming the technical and physical challenges still preventing TRCD from being applied to the X-ray domain, including the intrinsic weakness of the CD signal [90,91,92]. Using an X-ray probe would permit us to follow the temporal change in CD of a molecular target throughout, e.g., a chemical reaction [83].

HHG is a highly nonlinear optical process [93,94] providing coherent XUV and soft X-ray radiation with attosecond durations [95]. The HHG emission spectrum is characterized by a rapid decrease in the intensity for the low-order harmonics, consistent with perturbation theory, by a plateau region, in which the intensity of harmonic peaks is nearly constant, and then by an abrupt cutoff, beyond which almost no harmonics are observed [94]. Emitted photons are characterized by a frequency $\omega_{n}=n\omega$, which is an integer multiple of the pulse of the incoming pulse.

**Figure 7:**
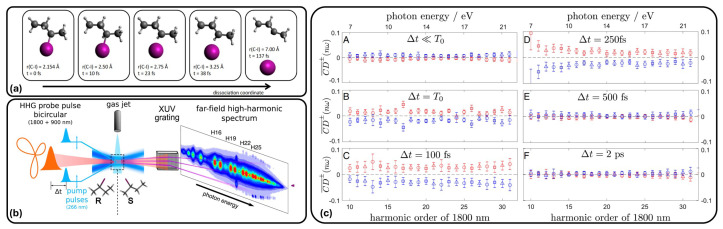
(**a**) Schematic representation of the photoinduced dissociation of the C-I bond in 2-iodobutane; (**b**) experimental setup; (**c**) ellipticity-averaged CD observed in 2-iodobutane as a function of the photon energy or harmonic order for different values of Δt. Adapted with permission from Ref. [83].

A pump–probe setup involving HHG pulses has been used to follow the light-induced dissociation of the chiral 2-iodobutane [83]. The molecule is first photoexcited at 266 nm, which triggers C-I bond breaking (panel (a)) of Figure 7). The sample is then irradiated with a given delay Δt by a two-color bicircular pulse, composed of the superposition of two intense pulses at 900 and 1800 nm with opposite helicities. The experimental setup is schematically reported in panel (b) of Figure 7; the HHG spectrum is given as a function of the harmonic order H$n=\frac{\omega_{n}}{\omega }$ (in the case of a two-color pulse, the reference frequency ω can be indistinctly one of the two defining the pulse). The idea is to follow the time evolution of chirality during the dissociation, since the bond breaking induces a change in chirality of the molecular system. Panel (c) of Figure 7 collects the average CD signal, ${\bar{CD}}^{\pm }$, as a function of the harmonic order H*n* for different values of the delay time Δt. The average concerns the fact that the experiment has been repeated for different helicities of the probe pulse, from linear to circular polarization; the ellipticity-resolved signal $CD^{\pm }$ has then been averaged to obtain ${\bar{CD}}^{\pm }$ around the circular polarization configurations, with opposite helicities. In other words, ${\bar{CD}}^{\pm }$ represents the sample response to a (nearly) clockwise or anticlockwise bicircular probe pulse; the superscript ± thus refers to a clockwise or counterclockwise probe pulse. The two sets of data in panel (c) of Figure 7 correspond to ${\bar{CD}}^{+}$ or ${\bar{CD}}^{-}$, i.e., the response of the sample, with a slight enantiomeric excess, to the clockwise or counterclockwise probe pulse; the two helicities interact differently with the sample, thus providing a different CD effect on the HHG spectrum. In the top left of panel (c) of Figure 7 ${\bar{CD}}^{\pm }$ is reported the absence of the pump pulse ($\Delta t<<T_{0}$, with $T_{0}$ being the time at which the sample is irradiated by the pump pulse); in this case, the CD signal of the unexcited molecules is rather small. The sample response is seen to be stronger with $\Delta t=T_{0}$, i.e., at the time when the pump pulse is switched on. Moreover, the CD response is the opposite with respect to the previous case. The induced chiral response persists up to around 250 fs, while at larger Δt values, it decays, indicating the formation of a nonchiral product.

These results are interpreted by considering the role of different channels in the ionization/recombination steps of the strong-field dynamics. Indeed, in the proposed model, supported by ab initio calculations, the induced chirality depends on the interplay between electric and magnetic dipole transitions occurring when the electron is ejected by the molecular target and behaves as a nearly free-charged particle in the continuum. Such an interplay generates the so-called crossing channels, where the electron removed from a certain molecular orbital recombines into a different one. Assuming that the ground and the first excited state of the cation are populated during the dynamics, which means that the electron is extracted either from HOMO or HOMO-1 of the neutral system, a cross-channel interaction can occur, providing a chiral-sensitive recombination of the electron with the parent ion. Details can be found in Ref. [83].

The other example reported here is that of TR-PECD with X-rays, recently employed to investigate the excited-state dynamics of fenchone enantiomers [85]. PECD is a well-known chiroptical effect exploited to study chiral systems in gas phase [79]. In PECD, a randomly aligned enantiomeric sample is ionized by a circularly polarized pulse; the intensity of the electron emission is highly asymmetric, thus being different in the forward and backward directions along the propagation axis of the ionizing pulse. This asymmetric chiral response reverses by changing the enantiomer or the handedness of the irradiated light. When applied to the electronic core, ECD provides a site- and chemical-specific chiral response, exploiting the well-known sensitivity of core orbitals to the surrounding environment [96,97].

**Figure 8:**
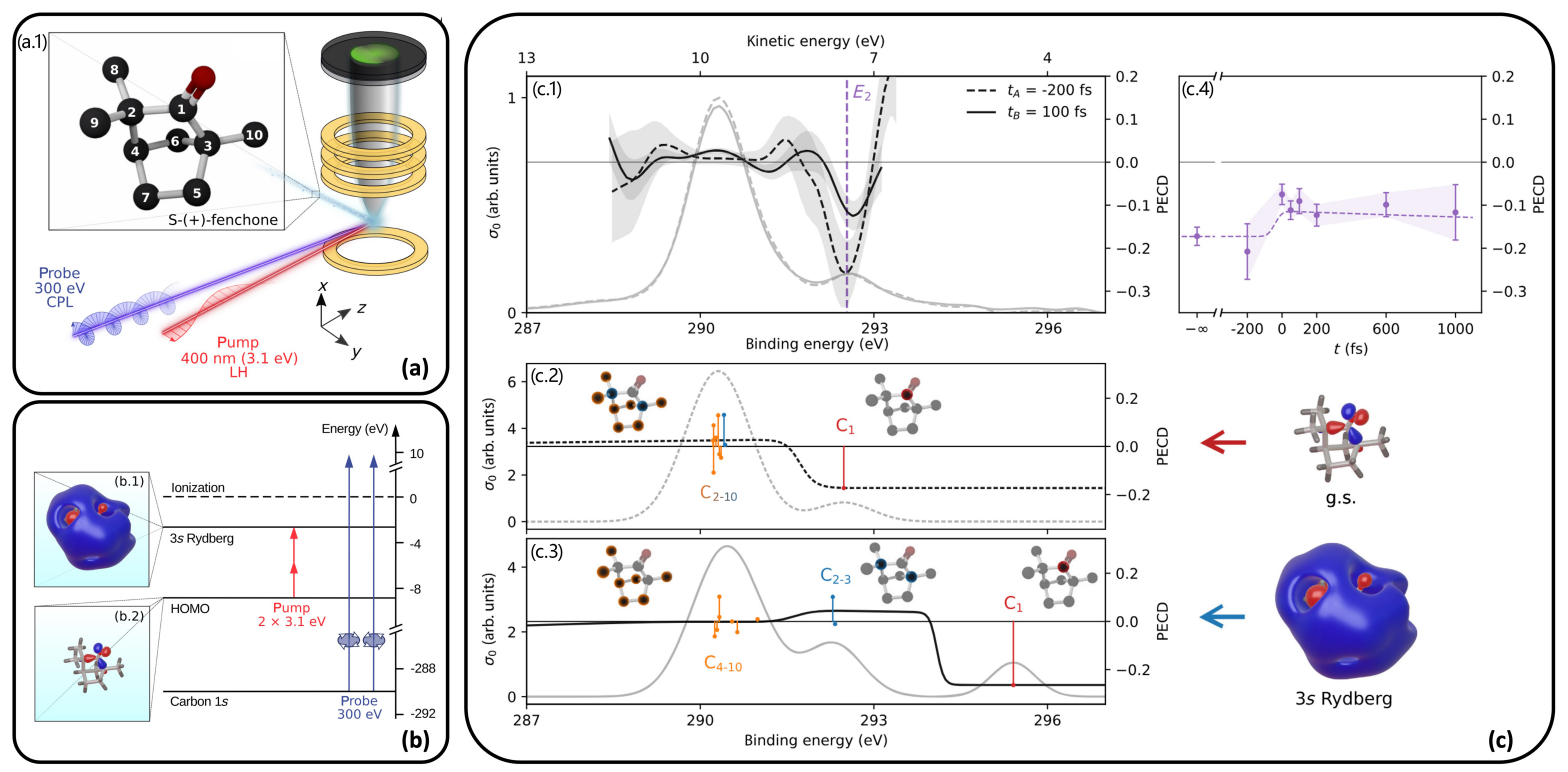
(**a**) Experimental setup for X-ray TR-PECD; (**b**) scheme of excitations involved in the experiment; (**c**) TR-PECD results. Adapted with permission from Ref. [85].

Access to the electronic/vibronic dynamics of a photoexcited molecule, as the key of enantiomeric sensitivity, is provided by TR-PECD. Indeed, TR-PECD on valence excitations in chiral molecules [87,88,89] allows probing the photoinduced ultrafast intra-molecular relaxation. TR-PECD with photoemission from core orbitals enriches the analysis of the chiral response with the atomic site sensitivity [85]. The experimental setup of Ref. [85] is shown in panel (a) of Figure 8. The sample of fenchone enantiomers is photoexcited by a linearly polarized ultrafast visible pump pulse with photon energy of ca. 3.1 eV, which induces an excitation from HOMO (see b.1 in Figure 8) into the diffuse 3*s* Rydberg orbital (LUMO+1, see b.2 in Figure 8) of fenchone. The probe pulse is a circularly polarized pulse with photon energy equal to 300 eV, generated within the Fermi free-electron laser facility. Pump–probe delays in the time window from −200 to 1000 fs have been used. This probe allows one to study the carbon *K* edge of fenchone. This process is summarized in panel (b) of Figure 8, where the two-photon pump excitation is highlighted. Experimental results on TR-PECD, supported by simulated data, are reported in panel (c) of Figure 8. In panel (c.1) of Figure 8 the experimental PECD at delay time −200 fs ($t_{a}$) and 100 fs ($t_{b}$) is reported, together with the photoelectron spectra at the same delays. A confidence interval to PECD is also given, as a shaded gray area. The binding energy $E_{2}=$ 292.53 eV corresponds to the carbonyl C 1*s* peak. With respect to the ground-state fenchone enantiomers, the excited sample is characterized by a larger (less negative) PECD. This result can be interpreted as originating from the different chiral asymmetry and binding energies of the 1*s* carbon states of the molecule in the excited 3*s* Rydberg state with respect to the ground-state molecule, due to the different local chiral environments experienced by the various carbon atoms upon excitation. This interpretation is made plausible by the photo-electron and PECD calculations, reported in panels (c.2) and (c.3) of Figure 8 for ground-state and excited fenchone, respectively. PECD from individual atoms, labelled according to the scheme in panel (a.1) of Figure 8, is presented as peaks. Theoretical PECD for ground-state fenchone at $E_{2}$ is negative and the only contribution is from C_1_, i.e., the carbonyl atom (panel (c.2)) of Figure 8. For the excited fenchone, the C_1_ no longer contributes at $E_{2}$, while a positive C_3_ contribution and a very small C_2_ contribution give a positive PECD at $E_{2}$. The measured TR-PECD at $E_{2}$ is reported as a function of the delay in panel (c.4) of Figure 8, together with the theoretical data, represented as a dashed line. The static PECD is given at −∞ on the time axis. TR-PECD at positive delay time values is less negative than that at −200 fs or the static one. The observed PECD at positive delays can be understood by a simple model assuming a 12.5% excitation probability; PECD values are therefore given by the weighted combination of the C_2_ and C_3_ 3*s* contributions with the residual ground-state C_1_ contribution. Details are found in Ref. [85].

In conclusion, the work of Ref. [85], briefly summarized here, represents a proof of concept of TR-PECD application in gas-phase molecular systems, as an enantiosensitive tool to study the chemical-specific and site-specific properties of a photoexcited chiral molecule.

XMCD is increasingly gaining recognition as a valuable technique in coordination chemistry and materials science. In this method, the difference in absorption of left- and right-circularly polarized X-rays by a magnetized sample is measured. This methodology provides quantitative information about the distribution of spin and orbital angular momenta and determines spin orientations from the sign of the XMCD signal [98,99,100,101,102,103,104,105,106,107]. In this context, time-resolved X-ray magnetic circular dichroism has been frequently applied to probe magnetic dynamics and phenomena in solids [25,26,108,109,110] and to study wave packet dynamics around the conical intersection in molecules [27]. In this kind of experiment, samples are usually irradiated by a UV-visible pump and probed by an attosecond circularly polarized X-ray pulse [27].

## 3. TRCD: Theoretical Methods

In the previous section, experimental techniques for TRCD and its applications in the study of different phenomena have been presented. The development of pump–probe setups to study the evolution of the CD signal in molecules allowed us to gain deep insights in the changes in and dynamics of the system under study [49,50,55]. These new technologies require suitable theoretical tools to study the time evolution of the CD signal, even originating from excited states of the sample. The accurate description of excited-state properties becomes of paramount importance when dealing with TRCD experiments. In order to follow the specific behavior of molecules in TRCD, it is necessary to account for the nuclear and electronic dynamics of the system, and for this reason, ab initio molecular dynamics [111] is considered a powerful tool to study these phenomena. When coupling among electronic states is non negligible, non-adiabatic molecular dynamics (NAMD) is employed [112,113]. In recent years, NAMD-based methods have been flourishing due to always newer and faster algorithms, which allow them to treat systems well over 100 atoms with reasonable computational cost [58]. Concurrently, many theoretical methods, based on linear and non-linear response [114], have been employed to study the properties of the excited states of the molecular system and to extract the CD spectra directly in a given geometry of the excited state. These methods do not account for the dynamic nature of the TRCD experiment, but they allow us to gain information regarding the excited-state properties of the system. In general, it is possible to compute the excited-state CD (ESCD) spectra by adopting a modified version of the Rosenfeld equation [115]:

(3) R=Im[〈L|m^→|K〉·〈K|μ^→|L〉].

where *R* is the rotatory strength and the two matrix elements, $\hat{m}$ and $\hat{\mu }$, are the magnetic and electric transition dipole moments between the excited states |K〉 and |L〉.

One of the theoretical challenges is precisely to define and compute the transition dipole moments of Equation (3).

### 3.1. Non-Adiabatic Molecular Dynamics

A key issue for theorists, not only for TRCD, is to be able to accurately describe the dynamics of molecular systems, considering electronic and nuclear motion, and the coupling between the two sets of degrees of freedom. Ab initio molecular dynamics is the ideal tool [111], where nuclei evolve in time classically, following forces computed at ab initio level of theory, within the Born–Oppenheimer (BO) approximation.

The most promising computational approach to studying non-adiabatic processes, frequently occurring in photochemistry, is NAMD [116]. NAMD simulations provide observables such as quantum yields or fluorescence intensities directly, without needing prior knowledge of the mechanism. This is particularly advantageous for larger systems with strong couplings, where determining a reaction coordinate might be difficult or impossible. The most well-known method for NAMD is probably Fewest Switches Surface Hopping (FSSH) [112]. In FSSH, non-adiabatic effects are modeled by the trajectory “hopping” between these potential energy surfaces (PESs) of different excited states in a stochastic fashion [112,116,117]. This methodology has been employed to study many different phenomena [118,119,120,121], including TRCD [58]. More specifically, FSSH has been coupled to time-dependent density functional theory (TDDFT) for the electronic degrees of freedom, allowing the investigation of systems with more than 100 atoms [122,123]. The first linear response (LR) TDDFT-NAMD implementation was developed by Tapavicza and co-workers within the Tamm–Dancoff approximation (TDA) [113,124]. Details about the method are found in Refs. [113,116].

Tapavicza and et al. applied LR-TDDFT-FSSH to the TRCD along the photoinduced ring-opening reaction of provitamin D (Pro) in gas phase [58]. It has been shown, both experimentally and theoretically [125,126], that this molecule undergoes several stages of isomerization after the initial photoexcitation. The ring-opening reaction in Pro has been investigated with ultrafast TRCD by Meyer et al. with a 120 fs time resolution, as also reported in Section 2.1 [57]. In this study, the initial structures of Pro were obtained from a Boltzmann ensemble at room temperature, generated from BO molecular dynamics. The nuclear coordinates were then propagated using the forces calculated at the TDDFT level for the first excited state ($S_{1}$). The non-adiabatic coupling terms between the ground state $S_{0}$ and $S_{1}$ were calculated and the probability of the switch between states computed at each time step [58,112].

The TRCD spectra were generated by calculating the instantaneous CD spectrum at each time step and subtracting it from the static CD spectrum of the Pro parent structure. The assumption made in this work is that the absorption occurs only in the ground state; hence, the transitions between excited states are not considered. This assumption is made considering that transitions from $S_{1}$ to higher excited states occur at energies which are lower than that of the probe, and the relaxation to the ground state occurs in all trajectories within 2 picoseconds of simulation time. This way, only the trajectories switching to the ground state contribute to the TRCD signal, which is then calculated by averaging over the number of trajectories [58].

**Figure 9:**
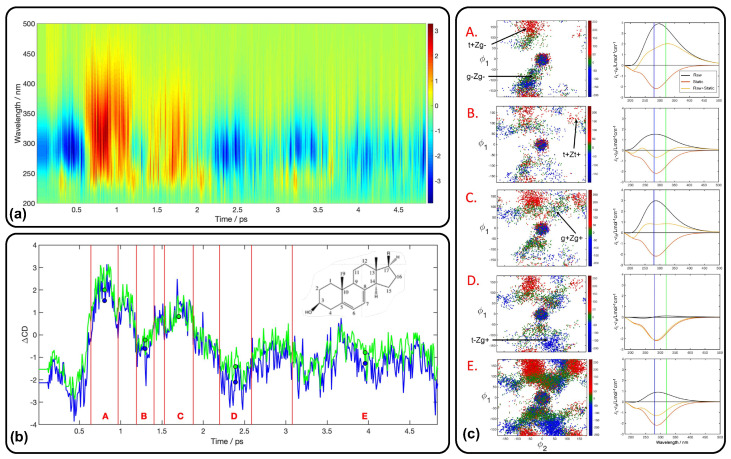
(**a**) Simulated TRCD spectrum along the photoinduced Pro ring-opening reaction. The color bar indicates the ΔCD signal in L/(mol·cm). (**b**) Traces along 280 (blue) and 300 (green) nm are taken from the broadband spectrum. Blue and green circles indicate the average ΔCD signal over the time windows A, B, C, D, and E, respectively. (**c**) In the left column, distribution of structures in the $\phi_{1}$/$\phi_{2}$ conformational space (dihedral angles defined by the atoms C10-C5-C6-C7 and C6-C7-C8-C9, respectively, of the structure displayed) is reported for the different time windows A, B, C, D, and E. In the right column, instantaneous CD spectrum Δϵ (black), static spectrum of Pro (red), and ΔCD (yellow) spectrum are reported, all averaged over the time interval. Adapted with permission from Ref. [58].

In panel (a) of Figure 9, the simulated time-resolved CD spectrum is shown throughout the photoinduced Pro ring-opening reaction, while the traces along 280 and 300 nm, taken from the broadband spectrum, are displayed in panel (b). In each time interval (labeled as A, B, C, D, and E in panel (b)), the average of the differential CD signal, ΔCD, is shown as green (320 nm) and blue (280 nm) circles. The signal decay, which is observed in the first part of the TRCD spectrum in panel (b), is associated with the relaxation to the ground state of the system and consequent ring-opening mechanism. The distribution of conformers for each time window is displayed in the left column of panel (c), while the instantaneous CD spectrum at each interval is displayed in the right column. These spectra allow following the isomerization mechanism after the photoinduced ring opening [58].

As mentioned above, the CD spectra have been computed by taking into account only transitions involving the ground state, which is a fair approximation considering the lower energy of the probe, as mentioned above, and the relaxation time of the system [58]. However, in order to interpret TRCD experiments, the computation of CD spectra involving transitions from the excited state is, in general, a necessary calculation. Over the years, methods based on linear and non-linear response have been developed to compute CD spectra from an excited molecule. The next section provides an overview of these methods and highlights their applications.

### 3.2. Excited-State CD Spectra

The transition dipole moments between excited states, which appear in Equation (3), naturally arise as part of the quadratic response (QR) and may be obtained by studying quadratic and cubic response functions [127,128,129]. Rizzo et al. [130], for instance, calculated the electric and magnetic transition dipole moments, in order to determine the excited-state rotatory strength (Equation (3)), from the calculation of the double residue of a quadratic response function. In this work, the authors computed the ESCD spectra of methyloxirane and binol. In the case of methyloxirane, the choice of the exchange-correlation (XC) functional causes significant changes in the spectral behavior for the three highest excited states, while a one-to-one correspondence is observed for the first five states. For binol, the rotatory strengths are strongly influenced by the electron correlation and the choice of the density functional [130]. Similarly, Schmid and coworkers performed experiments on the structure–function relationship of binol and two bridged derivatives, combining femtosecond TRCD and quantum chemical calculation [55]. In this work, the authors computed ESCD spectra for the four lowest-energy excited states of binol at some representative stationary points of their PES using the QR formalism [55]. Extensive details regarding the experimental part of this work are reported in Section 2.1. The coupled cluster response function methodology (CC-RSP) [131,132,133] has also been employed to compute ESCD spectra. CC-RSP is based on the QR formalism in the context of coupled cluster theory. We refer the interested reader to Ref. [132] for extensive details regarding the form of these specific response functions. This method was used to calculate the CD spectra of (1R)-Norcamphor by Andersen et al.; in this work, the authors compared the results with the spectra calculated at the Algebraic Diagrammatic Construction (ADC), Equation of Motion-Coupled Cluster (EOM-CC), and TDDFT level [134].

However, due to the high computational cost of QR-based methods, they are limited in the size of systems they can study. To address this restraint, approximations and methods based on linear response formalism have been developed. Among the theoretical approaches to study excited-state properties of molecular systems, LR-TDDFT is one of the most employed [135,136,137,138]. In the context of LR-TDDFT, many different approaches have been adopted. Mikhailov et al. proposed the application of TDA after the resolution of the linear response equations to calculate the transition moments between excited states [139]. In this work, the coupled electronic oscillator (CEO) [140] approach is used as a basis for the theoretical development of the formalism. CEO represents an alternative formulation of time-dependent perturbation theory for excited states, a representation based on the density matrix, which is propagated according to Heisenberg’s equation of motion. In this formalism, electronic states with double excitations are explicitly included. CEO provides results comparable to those from quadratic-response TDDFT [141].

Another approach was proposed by Segatta et al., who employed a pseudo-wavefunction TDDFT formalism [142]. In this context, the dynamical orbital relaxation effects are neglected, and the amplitudes obtained from LR-TDDFT are used to build the excited-state couplings and transition dipole moments, including up to single excitations.

In the pseudo-wavefunction formalism, the electronic excited-state pair |K〉, |L〉 is defined as:

(4) $$|K\rangle \equiv ({\hat{X}}^{k}+{\hat{X}}^{k}{\hat{X}}^{L}{\hat{Y}}^{L})|\Phi_{KS}\rangle ,$$

(5) $$|L\rangle \equiv ({\hat{X}}^{l}+{\hat{X}}^{l}{\hat{X}}^{k}{\hat{Y}}^{k})|\Phi_{KS}\rangle$$

where $|\Phi_{KS}\rangle$ is the reference (singly excited) Kohn–Sham determinant, and $\hat{X}$ and $\hat{Y}$ are the excitation operators:

(6) $${\hat{X}}^{K}=\sum_{ia}X_{i,K}^{a}{\hat{a}}_{a}^{†}{\hat{a}}_{i}$$

(7) $${\hat{Y}}^{K}=\sum_{ia}Y_{i,K}^{a}{\hat{a}}_{a}{\hat{a}}_{i}^{†}$$

$a^{†}$ and *a* are the creation and annihilation operators, respectively, and $X_{ia}^{K}$ and $Y_{ia}^{K}$ are the amplitudes obtained from the LR-TDDFT calculation using a Casida scheme [143]. Similar expressions are given for ${\hat{X}}^{L}$ and ${\hat{Y}}^{L}$. *i* and *a* run over the number of occupied and virtual molecular orbitals, respectively.

The electric transition dipole moments between excited states are consequently defined as [142]:

(8) $${\vec{\mu }}_{KL}=\sum_{ijab}\left(X_{i,K}^{a}X_{j,L}^{b}-Y_{i,K}^{a}Y_{j,L}^{b}\right)\left({\vec{\mu }}_{ab}\delta_{ij}-\delta_{ab}{\vec{\mu }}_{ij}\right),$$

with ${\vec{\mu }}_{ab}$ and ${\vec{\mu }}_{ij}$ being the transition dipole moments between virtual *a* and *b*, and occupied *i* and *j* molecular orbitals, respectively. In this work, this approach has been employed for obtaining the electric transition dipole moments. However, it has not been extended yet to the calculation of ESCD spectra; in the next section, future developments and perspectives are presented and discussed.

A similar method, which employed the same LR-TDDFT formalism in the construction of the transition dipole moments, has been recently presented in Refs. [144,145]. In these works, the authors computed the electric and magnetic transition dipole moments between excited states |K〉 and |L〉: [145]

(9) $$\langle L|\vec{\hat{\mu }}|K\rangle =\left(\begin{array}{cc} X^{L} & Y^{L} \end{array}\right)\left(\begin{array}{cc} \vec{\hat{\mu }} & 0\\ 0 & \vec{\hat{\mu }} \end{array}\right)\left(\begin{array}{c} X^{K}\\ Y^{K} \end{array}\right)=\left(X^{L}\vec{\hat{\mu }}X^{K}+Y^{L}\vec{\hat{\mu }}Y^{K}\right)$$

(10) $$\langle L|\vec{\hat{m}}|K\rangle =\left(\begin{array}{cc} X^{L} & Y^{L} \end{array}\right)\left(\begin{array}{cc} \vec{\hat{m}} & 0\\ 0 & \vec{\hat{m}} \end{array}\right)\left(\begin{array}{c} X^{K}\\ Y^{K} \end{array}\right)=\left(X^{L}\vec{\hat{m}}X^{K}-Y^{L}\vec{\hat{m}}Y^{K}\right).$$

These two equations provide a general formulation in the context of LR-TDDFT for calculating excited-state properties, and then can be rewritten in the molecular basis. The minus sign on the right-hand side of Equation (10) derives from the antisymmetric magnetic dipole operator $\vec{\hat{m}}$, and from the different action of $X^{L(K)}$ and $Y^{L(K)}$ on $\vec{\hat{m}}$. These equations generally work regardless of the specific type of response computed, and they have been recently used in time-domain calculations [144,146,147]. In Ref. [144], the authors employed Equations (9) and (10) to calculate the ESCD spectra of methyloxirane. Details about this work will be given in the next section.

The methodologies presented here combine low computational costs and reliable approximations for determining excited-state properties, showing great potential for the calculation of ESCD spectra.

Different theoretical approaches have been proposed as an alternative to TDDFT. Recently, the ADC scheme and CC-based approaches have been employed for the calculation of ESCD spectra to interpret TRCD experiments [134,148]. Green’s many-body function theory can be used to derive ADC for the polarization propagator Π in electronic excited states [149]. Π is applied to the time-dependent ground-state wavefunction and propagates the time-dependent density fluctuations of the many-body system [150,151]. In practice, Π is approximated in terms of perturbation theory expansion [149,152]. The level of accuracy of ADC is then related to this expansion, with ADC(n) indicating the perturbation order used to define Π. Accuracy of the computed excitation energies and transition amplitudes improve with an increase in the order of the perturbation theory [152,153]. In this context, a multi-reference generalization of this scheme was employed by Sokolov et al. [154] to study, for instance, excited electronic states. Another formulation of the ADC schemes exists in the so-called Intermediate State Representation (ISR) [155,156,157,158]. The concept of ISR can be used to obtain expressions for any generic operator and the corresponding expectation value, including the electric and magnetic dipole operators [152].

**Figure 10:**
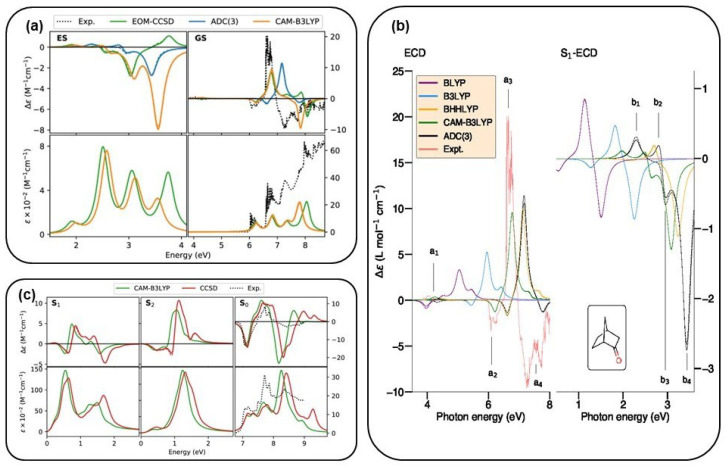
(**a**) S_1_ excited-state and ground-state ECD (upper panels) and absorption (bottom panels) spectra of (R)-Norcamphor at the EOM-CCSD, TDDFT/CAM-B3LYP, and ADC(3) (from Ref. [148]) levels are reported. (**b**) S_1_ (left) and ground-state (right) ECD spectra of (R)-Norcamphor computed at a DFT level with different exchange-correlation functionals as well as the ADC(3)/d-aug-cc-pVDZ results are displayed. The experimental ground-state ECD spectrum (red line) from Ref. [168] is given for comparison. (**c**) Calculated absorption (bottom panel) and CD (upper panel) spectra for S_0_, S_1_, and S_2_ of (R)-Methyloxirane with EOM-CC and TDDFT/CAM-B3LYP both with the d-aug-cc-pVDZ basis set are reported. Panels (**a**,**c**) are adapted from Ref. [148]. Panel (**b**) is adapted with permission from Ref. [134]. Copyright © 2022 American Chemical Society.

Authors of Ref. [148] used a perturbation scheme up to the third order (ADC(3)) and its second-order ISR to compute ESCD spectra for (1R)-camphor, (1R)-fenchone, (1R)-norcamphorm, and (R)-binol, then compared these to those obtained at the QR-DFT level [148]. In Figure 10, in panel (b), the ground-state CD and S_1_ ESCD spectra of (1R)-norcamphor are reported. In this work, the authors compared their results at the TDDFT level with different XC functionals as well as at the ADC(3) level using the d-aug-cc-pVDZ basis set. The spectra computed at the two theoretical levels are quite similar, despite a slight energy shift of about 0.4 eV. The consistency between these two different theoretical approaches in describing the ESCD spectrum of (1R)-norcamphor, along with their established precision in ground-state ECD analysis, proves the reliability of this ADC scheme for computing ESCD spectra of organic molecules [148].

A further option to compute ESCD spectra is the EOM-CC method, which has been devised to study excited-state properties of molecular systems [159,160,161,162]. It is a reliable and widely used theoretical tool for simulating optical properties. The range of techniques that can be addressed using EOM-CC is extensive and continually growing [163,164,165,166], encompassing both linear and nonlinear regimes. EOM-CC theory lays its foundations on the definition of an EOM operator, which acts on the CC wavefunction [159]. Within the non-Hermitian EOM-CC theory, the left and right eigenfunctions of $\hat{H}$ are not complex conjugates of each other. For this reason, it is necessary to define left and right EOM operators, $\hat{R}$ and $\hat{L}$. These two access a specific section of the Fock space, and their form depends on the target state n and the type of excitation to trigger [134,165,167]. In the context of the non-Hermitian CC theory, the right ${\langle \hat{A}\rangle }^{fn}$ and left ${\langle \hat{A}\rangle }^{nf}$ transition moments of a generic operator $\hat{A}$ between different states are not equal. As a result, the Rosenfeld equation needs to be explicitly symmetrized with respect to complex conjugation [134]. In the work of Andersen and co-workers [134], the authors calculated excited-state spectra for methyloxyrane and norcamphor using the EOM-CC method, by determining the transition electric and magnetic dipole moments between excited states with the reported formalism. In panels (a) and (c) of Figure 10, the ground-state and excited-state CD spectra for (1R)-Norcamphor and methyloxirane are reported and compared to those computed at other theoretical levels. In both cases, the shapes of the CD spectra are similar among the different methods employed, and only at high energy (above 3.5 eV) is it possible to detect some differences, as can be observed in the (1R)-Norcamphor case [134].

An intriguing study that integrates both experimental and theoretical approaches is detailed in Ref. [65]. The authors conducted a TRCD experiment, as outlined in Section 2.1, and also calculated the TRCD spectra of a squarine polymer. This was achieved by combining quantum chemical calculations on the isolated squaraine chromophore with a Frenkel exciton model for the excitonic system [65], thus going beyond the single-molecule response. The signal has been decomposed into ground-state bleaching (GBS), stimulated emission (SE), and excited-state absorption (ESA).

**Figure 11:**
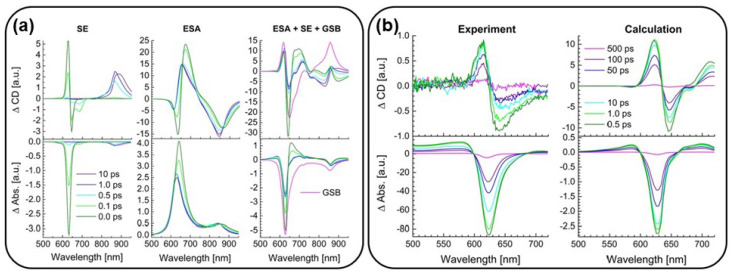
(**a**) ΔCD (top) and TA (bottom) spectra of squeezed helices. The contributions from SE, ESA, and GSB with their cumulative effect are displayed, respectively, in the left, middle, and right columns. (**b**) Comparison between the experimental and theoretical spectra for the squeezed helix; TRCD (top) and TA (bottom). The delay times between pump and probe pulse are given in the figure legend. Reproduced with permission from Ref. [65].

The squaraine monomer was studied using quantum chemical calculations with TDDFT, the CAM/B3LYP XC functional, and the 6–31 G** basis set. The authors were able to compute the ΔCD spectra to compare the theoretical and experimental data. In Figure 11 in panel (a), for instance, the TRCD and transient-absorption (TA) spectra of squeezed helices and their decomposition are reported with different time delays between the pump and the probe. The full TRCD spectrum is instead compared to the experimental results in panel (b) showing good qualitative agreement between theory and experiment. From these theoretical results, the authors predicted a pronounced TRCD and TA signals caused by SE from the high-energy exciton states in the range of 600–650 nm with time delays in the 100 fs window. It was also possible to detect strong TRCD signals of the low-energy exciton states around 850 nm, which were not visible in the TA measurements.

## 4. Discussion and Outlook

In this section, we focus on modeling TRCD, with the idea of discussing the potential of ab initio methods in the time-domain to reduce the gap with experimental conditions by efficiently computing ESCD spectra. The theoretical treatment of TRCD at short timescales involves the accurate description of molecular properties in excited electronic states, as described above. There are two main aspects we want to quickly overview here:

1. coupling techniques such as NAMD, which explore excited-state PESs, with approaches that compute ESCD spectra, with explicit focus on the population of excited states over time;
2. using the time-resolved version of methods such as LR-TDDFT to easily calculate ESCD in a general way, once an excited-state geometry is provided (from dynamics or geometry optimization).

Let us limit the discussion here to the TDDFT world, which explains why, in Section 3, we have given space to those methods, with detailed formulas. Concerning point 1, we have reported in Section 3.1 the work of Ref. [58] about the ring opening of provitamin D by using LR-TDDFT-FSSH. In that case, the CD spectra have been computed from the electronic ground state. To the best of our knowledge, a combined theoretical approach computing the ESCD spectra along a photoinduced molecular-dynamics pathway has not yet been presented in the literature. Such an approach could be used to simulate TRCD experiments, such as the photoloysis of MbCO [38,39,49] and the chiroptical changes in the binol enantiomers [55].

Regarding point 2, it is worth mentioning that the TDDFT pseudo-wavefunction formalism from Ref. [142] (Section 3.2) does not account for magnetic dipole moments yet, and consequently, it cannot be currently applied to study the CD signal from excited states. On the other hand, an example of the ESCD spectrum using the propagation of the time-dependent electronic wavefunction through the Time-Dependent Schrödinger Equation (TDSE) is reported in Ref. [144], as anticipated in Section 3.2. In this theoretical framework, the time-dependent electronic wavefunction $|\Psi (t_{el})\rangle$ is defined as the linear combination of the eigenstates of the field-free electronic Hamiltonian ${\hat{H}}_{0}$

(11) $$|\Psi \left(t_{el}\right)\rangle =\sum_{K=0}^{N_{\mathrm{states}}-1}C_{K}\left(t\right)|K\rangle ,$$

and then propagated according to the TDSE

(12) $$i\frac{d}{dt_{el}}|\Psi (t_{el})\rangle =\hat{H}\left(t_{el}\right)|\Psi (t_{el})\rangle \;\hat{H}\left(t_{el}\right)={\hat{H}}_{0}-\vec{\hat{\mu }}\cdot {\vec{E}}_{ext}\left(t_{el}\right).$$

In Equation (11) $C_{K}\left(t_{el}\right)$ are time-dependent coefficients, and |K〉 is the *K*-th eigenstate of the system, with eigenvalue $E_{K}$. These eigenstates are the Kohn–Sham DFT ground state and the ($N_{\mathrm{states}}-$1) TDDFT eigenstates in the singly excited ansatz [147,169]. In TDSE, the time-dependent term of the Hamiltonian is provided by the interaction term (in length gauge) $-\vec{\hat{\mu }}\cdot {\vec{E}}_{ext}\left(t_{el}\right)$, where ${\vec{E}}_{ext}\left(t_{el}\right)$ is the electric field of the external pulse, which plays the role of the CD probe. We explicitly refer to the time for electronic dynamics, i.e., $t_{el}$, to avoid confusion with the time *t* of a TRCD experiment. Using an explicit pulse in the calculations gives direct control over the probe properties, such as having a broadband or monochromatic light, the temporal duration, or the polarization.

The CD spectrum is computed starting from the induced magnetic dipole $\Delta \vec{m}\left(t_{el}\right)$, defined as the difference between the time-dependent magnetic moment $\vec{m}\left(t_{el}\right)$ at time $t_{el}$, and that at initial time ($t_{el}=0$), $\vec{m}\left(0\right)$, with $\vec{m}\left(t_{el}\right)$:

(13) $$\vec{m}\left(t_{el}\right)=\sum_{K,L}C_{L}^{*}\left(t_{el}\right)C_{K}\left(t_{el}\right)\langle L|\vec{\hat{m}}|K\rangle .$$

The time-dependent magnetic dipole moment of Equation (13), and thus $\Delta \vec{m}\left(t_{el}\right)$, require the calculation of the transition magnetic dipole between excited states, according to Equation (10) of Section 3.2. The CD spectrum is then obtained as proportional to the Fourier transform of $\Delta \vec{m}\left(t_{el}\right)$. Details can be found in Ref. [144]. In time-domain calculations, the Rosenfeld equation is therefore not used, including its extension to ESCD (Equation (3)).

**Figure 12:**
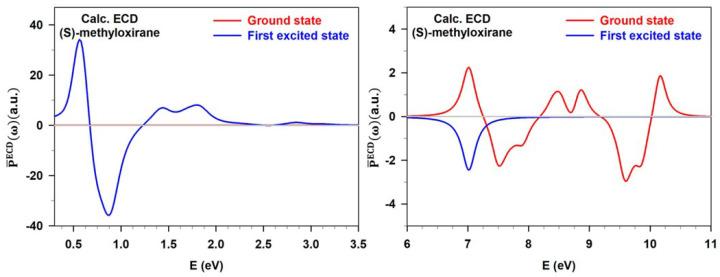
Calculated ECD spectra of (S)−methyloxirane considering the ground state (solid red lines) and the first excited state (solid blue lines). In the right panel, the energy window up to 3.5 eV is highlighted, and in the left, the energy window between 6 and 11 eV. Adapted with permission from Ref. [144]. Copyright © 2023, AIP.

In this work, the authors computed the first excited state ECD spectra of S-methyloxirane, imposing as an initial condition for the electronic dynamics that the population of the first excited state is $|C_{1}\left(t_{el}\right){|}^{2}=1$ on the optimized ground-state geometry, with $|C_{K}\left(t_{el}\right){|}^{2}$ being the population of the electronic state |K〉 at time $t_{el}$. This situation is the equivalent of a vertical transition from the ground state to the first excited state, with no relaxation. The spectra are reported in Figure 12. The chiral features observed for this molecule are heavily dependent on the initial condition, i.e., the initial population. Visible/UV contributions characterizing the ground-state CD spectrum are still present, but the most important peaks lie now between 0.5 and 2 eV.

The approach just described based on electronic TDSE has not yet been applied to the calculation of ESCD from optimized geometries in an excited state. However, the method is general and does not require any ad hoc modifications in the time-domain part; in fact, the TDSE would be solved using eigenergies and transition dipoles as ingredients that come from a TDDFT simulation on an excited state. Extension of the work in Ref. [144] could therefore represent a next step towards simulating TRCD experiments, together with combining NAMD and electronic TDSE in an integrated computational protocol.

A concrete idea to combine NAMD and time-domain TDSE is given in the following. What is interesting is that, based on the analysis of the populations of excited states from FSSH-based NAMD simulations (see Section 3.1), the calculation of ESCD spectra could exactly reproduce the electronic structure at time *t* and with that specific geometry. In fact, TDSE propagation for electronic degrees of freedom needs initial conditions, i.e., at time $t_{el}$ = 0, with $t_{el}$ being the instant at which the electronic dynamics starts. In this case, the initial populations would be directly given by the information obtained from the NAMD simulation. Of course, the ESCD spectrum will be given as an average over the FSSH trajectories for each time snapshot (or time interval). The energies and transition dipoles (electronic and magnetic) would be extracted from TDDFT simulations in the frequency domain on the reference geometry.

The simplest case is given by a system that only populates one excited electronic state; one can then imagine that the differential experimental spectrum, i.e., ΔCD, originates exclusively from the properties of a single excited state. If, on the other hand, due to non-adiabatic couplings, more than one excited state is appreciably populated at a certain time *t*, one can dissect the various contributions to the ΔCD by running TDSE simulations with different initial conditions, and possibly different parameters.

The electronic TDSE has been recently interfaced to the AMS [170] quantum chemistry package [144,145,147], which provides eigenenergies and transition dipole moments in the Slater atomic basis, and then a homemade interface transforms them in order to give the full matrix of the electric and magnetic dipole moment in the space of electronic states, including the $\langle L|\vec{\hat{\mu }}|K\rangle$ and $\langle L|\vec{\hat{m}}|K\rangle$ elements.

These suggestions for theoretical developments for TRCD do not require new methods or alternative formulations, but are based on the simple observation that already-existing ingredients, such as NAMD and electronic TDSE, can be successfully combined to provide a tool suitable for the interpretation of experimental TRCD data and ready to explore novel routes of investigation in the field of fast and ultrafast CD.

## Data Availability

No new data were created or analyzed in this study. Data sharing is not applicable to this article.

## References

[1] Pelayo J.J., Valencia I., García A.P., Chang L., López M., Toffoli D., Stener M., Fortunelli A., Garzón I.L. (2018). Chirality in bare and ligand-protected metal nanoclusters. Adv. Phys. X.

[2] Gray L.E., Ostby J., Furr J., Price M., Veeramachaneni D.R., Parks L. (2000). Perinatal exposure to the phthalates DEHP, BBP, and DINP, but not DEP, DMP, or DOTP, alters sexual differentiation of the male rat. Toxicol. Sci..

[3] Kim J.H., Scialli A.R. (2011). Thalidomide: The tragedy of birth defects and the effective treatment of disease. Toxicol. Sci..

[4] Brooks W.H., Guida W.C., Daniel K.G. (2011). The significance of chirality in drug design and development. Curr. Top. Med. Chem..

[5] Müller C., Bauer A., Bach T. (2009). Licht-getriebene enantioselektive Organokatalyse. Angew. Chem..

[6] Kameta N., Masuda M., Shimizu T. (2015). Qualitative/chiral sensing of amino acids by naked-eye fluorescence change based on morphological transformation and hierarchizing in supramolecular assemblies of pyrene-conjugated glycolipids. Chem. Commun..

[7] Frank F.C. (1953). On spontaneous asymmetric synthesis. Biochim. Biophys. Acta.

[8] Budin I., Szostak J.W. (2010). Expanding roles for diverse physical phenomena during the origin of life. Annu. Rev. Biophys..

[9] Meierhenrich U.J. (2013). Amino acids and the asymmetry of life. Eur. Rev..

[10] Pavlov V.A., I Klabunovskii E. (2014). Homochirality origin in nature: Possible versions. Curr. Org. Chem..

[11] Berova N., Nakanishi K., Woody R.W. (2000). Circular Dichroism: Principles and Applications.

[12] Stephens P.J. (1985). Theory of vibrational circular dichroism. J. Phys. Chem..

[13] Riehl J.P., Richardson F.S. (1986). Circularly polarized luminescence spectroscopy. Chem. Rev..

[14] Kuball H.G., Worsfold P., Poole C., Townshend A., Miró M. (2013). Chiroptical Analysis. Encyclopedia of Analytical Science.

[15] Berova N., Di Bari L., Pescitelli G. (2007). Application of electronic circular dichroism in configurational and conformational analysis of organic compounds. Chem. Soc. Rev..

[16] Fasman G.D. (2013). Circular Dichroism and the Conformational Analysis of Biomolecules.

[17] Serber R. (1932). The Theory of the Faraday Effect in Molecules. Phys. Rev..

[18] Buckingham A.D., Stephens P.J. (1966). Magnetic Optical Activity. Annu. Rev. Phys. Chem..

[19] Mason W.R. (2007). A Practical Guide to Magnetic Circular Dichroism Spectroscopy.

[20] Piepho S.B., Schatz P.N. (1983). Group Theory in Spectroscopy: With Applications to Magnetic Circular Dichroism.

[21] Elove G.A., Chaffotte A.F., Roder H., Goldberg M.E. (1992). Early steps in cytochrome c folding probed by time-resolved circular dichroism and fluorescence spectroscopy. Biochemistry.

[22] Jones C.M., Henry E.R., Hu Y., Chan C.K., Luck S.D., Bhuyan A., Roder H., Hofrichter J., Eaton W.A. (1993). Fast events in protein folding initiated by nanosecond laser photolysis. Proc. Natl. Acad. Sci. USA.

[23] van der Laan G., Figueroa A.I. (2014). X-ray magnetic circular dichroism—A versatile tool to study magnetism. Coord. Chem. Rev..

[24] Fan T., Grychtol P., Knut R., Hernandez-Garcia C., Hickstein D.D., Zusin D., Gentry C., Dollar F.J., Mancuso C.A., Hogle C.W. (2015). Bright circularly polarized soft X-ray high harmonics for X-ray magnetic circular dichroism. Proc. Natl. Acad. Sci. USA.

[25] Stamm C., Kachel T., Pontius N., Mitzner R., Quast T., Holldack K., Khan S., Lupulescu C., Aziz E.F., Wietstruk M. (2007). Femtosecond modification of electron localization and transfer of angular momentum in nickel. Nat. Mater..

[26] Boeglin C., Beaurepaire E., Halté V., López-Flores V., Stamm C., Pontius N., Dürr H.A., Bigot J.Y. (2010). Distinguishing the ultrafast dynamics of spin and orbital moments in solids. Nature.

[27] Sun S., Gu B., Hu H., Lu L., Tang D., Chernyak V.Y., Li X., Mukamel S. (2024). Direct Probe of Conical Intersection Photochemistry by Time-Resolved X-ray Magnetic Circular Dichroism. J. Am. Chem. Soc..

[28] Bayley P. (1981). Fast kinetic studies with chiroptical techniques: Stopped flow circular dichroism and related methods. Prog. Biophys. Mol. Biol..

[29] Anson M., Martin S.R., Bayley P.M. (1977). Transient CD measurements at submillisecond time resolution—Application to studies of temperature-jump relaxation of equilibria of chiral biomolecules. Rev. Sci. Instrum..

[30] Ferrone F., Hopfield J., Schnatterly S. (1974). The measurement of transient circular dichroism: A new kinetic technique. Rev. Sci. Instrum..

[31] Laouer K., Schmid M., Wien F., Changenet P., Hache F. (2021). Folding Dynamics of DNA G-Quadruplexes Probed by Millisecond Temperature Jump Circular Dichroism. J. Phys. Chem. B.

[32] Khuc M.T., Mendonça L., Sharma S., Solinas X., Volk M., Hache F. (2011). Measurement of circular dichroism dynamics in a nanosecond temperature-jump experiment. Rev. Sci. Instrum..

[33] Mendonça L., Steinbacher A., Bouganne R., Hache F. (2014). Comparative Study of the Folding/Unfolding Dynamics of Poly(glutamic acid) in Light and Heavy Water. J. Phys. Chem. B.

[34] Chen E., Wittung-Stafshede P., Kliger D.S. (1999). Far-UV Time-Resolved Circular Dichroism Detection of Electron-Transfer-Triggered Cytochrome c Folding. J. Am. Chem. Soc..

[35] Kuwajima K., Yamaya H., Miwa S., Sugai S., Nagamura T. (1987). Rapid formation of secondary structure framework in protein folding studied by stopped-flow circular dichroism. FEBS Lett..

[36] Thomas Y.G., Szundi I., Lewis J.W., Kliger D.S. (2009). Microsecond Time-Resolved Circular Dichroism of Rhodopsin Photointermediates. Biochemistry.

[37] Clinger J.A., Chen E., Kliger D.S., Phillips G.N.J. (2021). Pump–Probe Circular Dichroism Spectroscopy of Cyanobacteriochrome TePixJ Yields: Insights into Its Photoconversion. J. Chem. Phys. B.

[38] Milder S., Bjorling S., Kuntz I., Kliger D. (1988). Time-resolved circular dichroism and absorption studies of the photolysis reaction of (carbonmonoxy) myoglobin. Biophys. J..

[39] Chen E., Kliger D.S. (1996). Time-resolved near UV circular dichroism and absorption studies of carbonmonoxymyoglobin photolysis intermediates. Inorg. Chim. Acta.

[40] Björling S.C., Goldbeck R.A., Paquette S.J., Milder S.J., Kliger D.S. (1996). Allosteric Intermediates in Hemoglobin. 1. Nanosecond Time-Resolved Circular Dichroism Spectroscopy. Biochemistry.

[41] Lewis J.W., Goldbeck R.A., Kliger D.S., Xie X., Dunn R.C., Simon J.D. (1992). Time-resolved circular dichroism spectroscopy: Experiment, theory, and applications to biological systems. J. Phys. Chem..

[42] Goldbeck R.A., Kim-Shapiro D.B., Kliger D.S. (1997). Fast Natural and Magnetic Circular Dichroism Spectroscopy. Annu. Rev. Phys. Chem..

[43] Chen E., Goldbeck R.A., Kliger D.S. (2010). Nanosecond time-resolved polarization spectroscopies: Tools for probing protein reaction mechanisms. Methods.

[44] Hache F., Changenet P. (2021). Multiscale conformational dynamics probed by time-resolved circular dichroism from seconds to picoseconds. Chirality.

[45] Meyer-Ilse J., Akimov D., Dietzek B. (2013). Recent advances in ultrafast time-resolved chirality measurements: Perspective and outlook. Laser Photonics Rev..

[46] Changenet P., Hache F. (2023). Recent advances in the development of ultrafast electronic circular dichroism for probing the conformational dynamics of biomolecules in solution. Eur. Phys. J. Spec. Top..

[47] Lewis J., Tilton R., Einterz C., Milder S., Kuntz I., Kliger D. (1985). New technique for measuring circular dichroism changes on a nanosecond time scale. Application to (carbonmonoxy) myoglobin and (carbonmonoxy) hemoglobin. J. Phys. Chem..

[48] Xie X., Simon J.D. (1989). Picosecond time-resolved circular dichroism spectroscopy: Experimental details and applications. Rev. Sci. Instrum..

[49] Dartigalongue T., Hache F. (2005). Observation of sub-100ps conformational changes in photolyzed carbonmonoxy-myoglobin probed by time-resolved circular dichroism. Chem. Phys. Lett..

[50] Niezborala C., Hache F. (2006). Measuring the dynamics of circular dichroism in a pump-probe experiment with a Babinet-Soleil Compensator. J. Opt. Soc. Am. B.

[51] Dartigalongue T., Hache F. (2006). Time-resolved circular dichroism in carbonmonoxy-myoglobin: The central role of the proximal histidine. Chirality.

[52] Dartigalongue T., Niezborala C., Hache F. (2007). Subpicosecond UV spectroscopy of carbonmonoxy-myoglobin: Absorption and circular dichroism studies. Phys. Chem. Chem. Phys..

[53] Hache F., Khuc M.T., Brazard J., Plaza P., Martin M.M., Checcucci G., Lenci F. (2009). Picosecond transient circular dichroism of the photoreceptor protein of the light-adapted form of Blepharisma japonicum. Chem. Phys. Lett..

[54] Mendonça L., Hache F., Changenet-Barret P., Plaza P., Chosrowjan H., Taniguchi S., Imamoto Y. (2013). Ultrafast carbonyl motion of the photoactive yellow protein chromophore probed by femtosecond circular dichroism. J. Am. Chem. Soc..

[55] Schmid M., Martinez-Fernandez L., Markovitsi D., Santoro F., Hache F., Improta R., Changenet P. (2019). Unveiling excited-state chirality of binaphthols by femtosecond circular dichroism and quantum chemical calculations. J. Phys. Chem. Lett..

[56] Dartigalongue T., Hache F. (2005). Calculation of the circular dichroism spectra of carbon monoxy- and deoxy myoglobin: Interpretation of a time-resolved circular dichroism experiment. J. Chem. Phys..

[57] Meyer-Ilse J., Akimov D., Dietzek B. (2012). Ultrafast Circular Dichroism Study of the Ring Opening of 7-Dehydrocholesterol. J. Phys. Chem. Lett..

[58] Tapavicza E., Reutershan T., Thompson T. (2023). Ab Initio Simulation of the Ultrafast Circular Dichroism Spectrum of Provitamin D Ring-Opening. J. Phys. Chem. Lett..

[59] Trifonov A., Buchvarov I., Lohr A., Würthner F., Fiebig T. (2010). Broadband femtosecond circular dichroism spectrometer with white-light polarization control. Rev. Sci. Instrum..

[60] Scholz M., Morgenroth M., Cho M.J., Choi D.H., Oum K., Lenzer T. (2019). Ultrafast Broadband Transient Absorption and Circular Dichroism Reveal Relaxation of a Chiral Copolymer. J. Phys. Chem. Lett..

[61] Morgenroth M., Scholz M., Lenzer T., Oum K. (2020). Ultrafast UV–Vis Transient Absorption and Circular Dichroism Spectroscopy of a Polyfluorene Copolymer Showing Large Chiral Induction. J. Phys. Chem. C.

[62] Jespersen K.G., Beenken W.J.D., Zaushitsyn Y., Yartsev A., Andersson M., Pullerits T., Sundström V. (2004). The electronic states of polyfluorene copolymers with alternating donor-acceptor units. J. Chem. Phys..

[63] Morgenroth M., Scholz M., Cho M.J., Choi D.H., Oum K., Lenzer T. (2022). Mapping the broadband circular dichroism of copolymer films with supramolecular chirality in time and space. Nat. Commun..

[64] Gust D., Scholz M., Schumacher V., Mulatier J.C., Pitrat D., Guy L., Oum K., Lenzer T. (2024). Annealing temperature-dependent induced supramolecular chiroptical response of copolymer thin films studied by pump-modulated transient circular dichroism spectroscopy. Sci. Rep..

[65] Ress L., Malỳ P., Landgraf J.B., Lindorfer D., Hofer M., Selby J., Lambert C., Renger T., Brixner T. (2023). Time-resolved circular dichroism of excitonic systems: Theory and experiment on an exemplary squaraine polymer. Chem. Sci..

[66] Oppermann M., Bauer B., Rossi T., Zinna F., Helbing J., Lacour J., Chergui M. (2019). Ultrafast broadband circular dichroism in the deep ultraviolet. Optica.

[67] Gold J.S., Milder S.J., Lewis J.W., Kliger D.S. (1985). Transient circular dichroism of the luminescent state of Ru(bpy)32+. J. Am. Chem. Soc..

[68] Milder S.J., Gold J.S., Kliger D.S. (1988). Time-resolved circular dichroism of the lowest excited state of (*Δ*)-Ru(bpy)32+. Chem. Phys. Lett..

[69] Oppermann M., Spekowius J., Bauer B., Pfister R., Chergui M., Helbing J. (2019). Broad-Band Ultraviolet CD Spectroscopy of Ultrafast Peptide Backbone Conformational Dynamics. J. Phys. Chem. Lett..

[70] Mangot L., Taupier G., Romeo M., Boeglin A., Cregut O., Dorkenoo K.D.H. (2010). Broadband transient dichroism spectroscopy in chiral molecules. Opt. Lett..

[71] Eom I., Ahn S.H., Rhee H., Cho M. (2012). Single-Shot Electronic Optical Activity Interferometry: Power and Phase Fluctuation-Free Measurement. Phys. Rev. Lett..

[72] Hiramatsu K., Nagata T. (2015). Communication: Broadband and ultrasensitive femtosecond time-resolved circular dichroism spectroscopy. J. Chem. Phys..

[73] Preda F., Perri A., Réhault J., Dutta B., Helbing J., Cerullo G., Polli D. (2018). Time-domain measurement of optical activity by an ultrastable common-path interferometer. Opt. Lett..

[74] Nishiyama Y., Ishikawa S., Nagatani H. (2020). Phase-stable optical activity measurement by common-path spectral interferometry. Opt. Lett..

[75] Ghosh S., Herink G., Perri A., Preda F., Manzoni C., Polli D., Cerullo G. (2021). Broadband Optical Activity Spectroscopy with Interferometric Fourier-Transform Balanced Detection. ACS Photonics.

[76] Changenet P., Hache F. (2023). Artifact-free balanced detection for the measurement of circular dichroism with a sub-picosecond time resolution. Opt. Express.

[77] Niezborala C., Hache F. (2007). Excited-State Absorption and Circular Dichroism of Ruthenium(II) Tris(phenanthroline) in the Ultraviolet Region. J. Phys. Chem. A.

[78] Ayuso D., Ordonez A.F., Smirnova O. (2022). Ultrafast chirality: The road to efficient chiral measurements. Phys. Chem. Chem. Phys..

[79] Powis I. (2008). Photoelectron Circular Dichroism in Chiral Molecules. Adv. Chem. Phys..

[80] Cireasa R., Boguslavskiy A.E., Pons B., Wong M.C.H., Descamps D., Petit S., Ruf H., Thiré N., Ferré A., Suarez J. (2015). Probing molecular chirality on a sub-femtosecond timescale. Nat. Phys..

[81] Harada Y., Haraguchi E., Kaneshima K., Sekikawa T. (2018). Circular dichroism in high-order harmonic generation from chiral molecules. Phys. Rev. A.

[82] Baykusheva D., Wörner H.J. (2018). Chiral Discrimination through Bielliptical High-Harmonic Spectroscopy. Phys. Rev. X.

[83] Baykusheva D., Zindel D., Svoboda V., Bommeli E., Ochsner M., Tehlar A., Wörner H.J. (2019). Real-time probing of chirality during a chemical reaction. Proc. Natl. Acad. Sci. USA.

[84] Svoboda V., Ram N.B., Baykusheva D., Zindel D., Waters M.D.J., Spenger B., Ochsner M., Herburger H., Stohner J., Wörner H.J. (2022). Femtosecond photoelectron circular dichroism of chemical reactions. Sci. Adv..

[85] Faccialà D., Devetta M., Beauvarlet S., Besley N., Calegari F., Callegari C., Catone D., Cinquanta E., Ciriolo A.G., Colaizzi L. (2023). Time-Resolved Chiral X-Ray Photoelectron Spectroscopy with Transiently Enhanced Atomic Site Selectivity: A Free-Electron Laser Investigation of Electronically Excited Fenchone Enantiomers. Phys. Rev. X.

[86] Tikhonov D.S., Blech A., Leibscher M., Greenman L., Schnell M., Koch C.P. (2022). Pump-probe spectroscopy of chiral vibrational dynamics. Sci. Adv..

[87] Comby A., Beaulieu S., Boggio-Pasqua M., Descamps D., Légaré F., Nahon L., Petit S., Pons B., Fabre B., Mairesse Y. (2016). Relaxation Dynamics in Photoexcited Chiral Molecules Studied by Time-Resolved Photoelectron Circular Dichroism: Toward Chiral Femtochemistry. J. Phys. Chem. Lett..

[88] Beaulieu S., Comby A., Fabre B., Descamps D., Ferré A., Garcia G., Géneaux R., Légaré’ F., Nahon L., Petit S. (2016). Probing Ultrafast Dynamics of Chiral Molecules Using Time-Resolved Photoelectron Circular Dichroism. Faraday Discuss..

[89] Blanchet V., Descamps D., Petit S., Mairesse Y., Pons B., Fabre B. (2021). Ultrafast Relaxation Investigated by Photoelectron Circular Dichroism: An Isomeric Comparison of Camphor and Fenchone. Phys. Chem. Chem. Phys..

[90] Rouxel J.R., Kowalewski M., Mukamel S. (2017). Photoinduced molecular chirality probed by ultrafast resonant x-ray spectroscopy. Struct. Dyn..

[91] Zhang Y., Rouxel J.R., Autschbach J., Govind N., Mukamel S. (2017). X-ray circular dichroism signals: A unique probe of local molecular chirality. Chem. Sci..

[92] Rouxel J.R., Zhang Y., Mukamel S. (2019). X-ray Raman optical activity of chiral molecules. Chem. Sci..

[93] Corkum P.B. (1993). Plasma perspective on strong field multiphoton ionization. Phys. Rev. Lett..

[94] Lewenstein M., Balcou P., Ivanov M.Y., L’Huillier A., Corkum P.B. (1994). Theory of high-harmonic generation by low-frequency laser fields. Phys. Rev. A.

[95] Corkum P.B., Krausz F. (2007). Attosecond Science. Nat. Phys..

[96] Turchini S., Catone D., Zema N., Contini G., Prosperi T., Decleva P., Stener M., Rondino F., Piccirillo S., Prince K.C. (2013). Conformational Sensitivity in Photoelectron Circular Dichroism of 3-Methylcyclopentanone. ChemPhysChem.

[97] Ilchen M., Hartmann G., Rupprecht P., Artemyev A.N., Coffee R.N., Li Z., Ohldag H., Ogasawara H., Osipov T., Ray D. (2017). Emitter-Site-Selective Photoelectron Circular Dichroism of Trifluoromethyloxirane. Phys. Rev. A.

[98] Schuetz G., Wagner W., Wilhelm W., Kienle P., Zeller R., Frahm R., Materlik G. (1987). Absorption of circularly polarized x-rays in iron. Phys. Rev. Lett..

[99] Chen C., Idzerda Y., Lin H.J., Smith N., Meigs G., Chaban E., Ho G., Pellegrin E., Sette F. (1995). Experimental Confirmation of the X-ray Magnetic Circular Dichroism Sum Rules for Iron and Cobalt. Phys. Rev. Lett..

[100] Funk T., Deb A., George S.J., Wang H., Cramer S.P. (2005). X-ray magnetic circular dichroism—A high energy probe of magnetic properties. Coordin. Chem. Rev..

[101] Zamudio-Bayer V., Hirsch K., Langenberg A., Niemeyer M., Vogel M., Ławicki A., Terasaki A., Lau J.T., Von Issendorff B. (2015). Maximum Spin Polarization in Chromium Dimer Cations as Demonstrated by X-ray Magnetic Circular Dichroism Spectroscopy. Angew. Chem. Int. Ed..

[102] Wilhelm F., Sanchez J.P., Rogalev A. (2018). Magnetism of uranium compounds probed by XMCD spectroscopy. J. Phys. D Appl. Phys..

[103] Bar A.K., Kalita P., Singh M.K., Rajaraman G., Chandrasekhar V. (2018). Low-coordinate mononuclear lanthanide complexes as molecular nanomagnets. Coord. Chem. Rev..

[104] Pedersen K.S., Meihaus K.R., Rogalev A., Wilhelm F., Aravena D., Amoza M., Ruiz E., Long J.R., Bendix J., Clérac R. (2019). [UF_6_]^2−^: A Molecular Hexafluorido Actinide(IV) Complex with Compensating Spin and Orbital Magnetic Moments. Angew. Chem. Int. Ed..

[105] Kowalska J.K., Henthorn J.T., Van Stappen C., Trncik C., Einsle O., Keavney D., DeBeer S. (2019). X-ray Magnetic Circular Dichroism Spectroscopy Applied to Nitrogenase and Related Models: Experimental Evidence for a Spin-Coupled Molybdenum (III) Center. Angew. Chem. Int. Ed..

[106] Jungcharoen P., Pédrot M., Choueikani F., Pasturel M., Hanna K., Heberling F., Tesfa M., Marsac R. (2021). Probing the effects of redox conditions and dissolved Fe^2+^ on nanomagnetite stoichiometry by wet chemistry, XRD, XAS and XMCD. Environ. Sci. Nano.

[107] N’Diaye A., Bordage A., Nataf L., Baudelet F., Rivière E., Bleuzen A. (2022). Interplay between Transition-Metal K-edge XMCD and Magnetism in Prussian Blue Analogs. ACS Omega.

[108] Takubo K., Yamamoto K., Hirata Y., Yokoyama Y., Kubota Y., Yamamoto S., Yamamoto S., Matsuda I., Shin S., Seki T. (2017). Capturing ultrafast magnetic dynamics by time-resolved soft x-ray magnetic circular dichroism. Appl. Phys. Lett..

[109] Ishii Y., Yamasaki Y., Kozuka Y., Lustikova J., Nii Y., Onose Y., Yokoyama Y., Mizumaki M., ichi Adachi J., Nakao H. (2024). Microscopic evaluation of spin and orbital moment in ferromagnetic resonance. Sci. Rep..

[110] Emori S., Maizel R.E., Street G.T., Jones J.L., Arena D.A., Shafer P., Klewe C. (2024). Quantifying the orbital-to-spin moment ratio under dynamic excitation. Appl. Phys. Lett..

[111] Iftimie R., Minary P., Tuckerman M.E. (2005). Ab initio molecular dynamics: Concepts, recent developments, and future trends. Proc. Natl. Acad. Sci. USA.

[112] Tully J.C. (1990). Molecular dynamics with electronic transitions. J. Chem. Phys..

[113] Tapavicza E., Tavernelli I., Rothlisberger U. (2007). Trajectory Surface Hopping within Linear Response Time-Dependent Density-Functional Theory. Phys. Rev. Lett..

[114] McWeeny R. (1992). Methods of Molecular Quantum Mechanics.

[115] Schellman J.A. (1975). Circular dichroism and optical rotation. Chem. Rev..

[116] Tapavicza E., Bellchambers G.D., Vincent J.C., Furche F. (2013). Ab initio non-adiabatic molecular dynamics. Phys. Chem. Chem. Phys..

[117] Liu J., Neukirch A.J., Prezhdo O.V. (2014). Non-radiative electron–hole recombination in silicon clusters: Ab initio non-adiabatic molecular dynamics. J. Phys. Chem. C.

[118] Chapman S. (1992). The Classical Trajectory-Surface-Hopping Approach to Charge-Transfer Processes. Advances in Chemical Physics: State-Selected and State-To-State Ion-Molecule Reaction Dynamics, Part 2, Theory.

[119] Subotnik J.E., Jain A., Landry B., Petit A., Ouyang W., Bellonzi N. (2016). Understanding the surface hopping view of electronic transitions and decoherence. Annu. Rev. Phys. Chem..

[120] Malhado J.P., Bearpark M.J., Hynes J.T. (2014). Non-adiabatic dynamics close to conical intersections and the surface hopping perspective. Front. Chem..

[121] Barbatti M. (2011). Nonadiabatic dynamics with trajectory surface hopping method. Wiley Interdiscip. Rev. Comput. Mol. Sci..

[122] Furche F., Ahlrichs R. (2002). Adiabatic time-dependent density functional methods for excited state properties. J. Chem. Phys..

[123] Send R., Furche F. (2010). First-order nonadiabatic couplings from time-dependent hybrid density functional response theory: Consistent formalism, implementation, and performance. J. Chem. Phys..

[124] Tavernelli I., Curchod B.F., Rothlisberger U. (2009). On nonadiabatic coupling vectors in time-dependent density functional theory. J. Chem. Phys..

[125] Sofferman D.L., Konar A., Spears K.G., Sension R.J. (2021). Ultrafast excited state dynamics of provitamin D3 and analogs in solution and in lipid bilayers. J. Chem. Phys..

[126] Tapavicza E., Meyer A.M., Furche F. (2011). Unravelling the details of vitamin D photosynthesis by non-adiabatic molecular dynamics simulations. Phys. Chem. Chem. Phys..

[127] Sałek P., Vahtras O., Helgaker T., Ågren H. (2002). Density-functional theory of linear and nonlinear time-dependent molecular properties. J. Chem. Phys..

[128] Jansik B., Sałek P., Jonsson D., Vahtras O., Ågren H. (2005). Cubic response functions in time-dependent density functional theory. J. Chem. Phys..

[129] Gaw J., Handy N. (1985). Ab initio quadratic, cubic and quartic force constants for the calculation of spectroscopic constants. Chem. Phys. Lett..

[130] Rizzo A., Vahtras O. (2011). Ab initio study of excited state electronic circular dichroism. Two prototype cases: Methyl oxirane and R-(+)-1, 1^′^-bi (2-naphthol). J. Chem. Phys..

[131] Helgaker T., Coriani S., Jørgensen P., Kristensen K., Olsen J., Ruud K. (2012). Recent advances in wave function-based methods of molecular-property calculations. Chem. Rev..

[132] Koch H. (1990). Coupled cluster response functions. J. Chem. Phys..

[133] Christiansen O., Jørgensen P., Hättig C. (1998). Response functions from Fourier component variational perturbation theory applied to a time-averaged quasienergy. Int. J. Quantum Chem..

[134] Andersen J.H., Nanda K.D., Krylov A.I., Coriani S. (2022). Probing Molecular Chirality of Ground and Electronically Excited States in the UV–vis and X-ray Regimes: An EOM-CCSD Study. J. Chem. Theory Comput..

[135] Pescitelli G., Bruhn T. (2016). Good computational practice in the assignment of absolute configurations by TDDFT calculations of ECD spectra. Chirality.

[136] Bannwarth C., Seibert J., Grimme S. (2016). Electronic circular dichroism of [16]Helicene with simplified TD-DFT: Beyond the single structure approach. Chirality.

[137] Jang H., Kim N.J., Heo J. (2018). Benchmarking study on time-dependent density functional theory calculations of electronic circular dichroism for gas-phase molecules. Comput. Theor. Chem..

[138] Bannwarth C., Grimme S. (2014). A simplified time-dependent density functional theory approach for electronic ultraviolet and circular dichroism spectra of very large molecules. Comput. Theor. Chem..

[139] Mikhailov I.A., Tafur S., Masunov A.E. (2008). Double excitations and state-to-state transition dipoles in *π*-*π** excited singlet states of linear polyenes: Time-dependent density-functional theory versus multiconfigurational methods. Phys. Rev. A.

[140] Tretiak S., Mukamel S. (2002). Density matrix analysis and simulation of electronic excitations in conjugated and aggregated molecules. Chem. Rev..

[141] Nayyar I.H., Masunov A.E., Tretiak S. (2013). Comparison of TD-DFT methods for the calculation of two-photon absorption spectra of oligophenylvinylenes. J. Chem. Phys. C.

[142] Segatta F., Russo M., Nascimento D.R., Presti D., Rigodanza F., Nenov A., Bonvicini A., Arcioni A., Mukamel S., Maiuri M. (2021). In Silico Ultrafast Nonlinear Spectroscopy Meets Experiments: The Case of Perylene Bisimide Dye. J. Chem. Theory Comput..

[143] Casida M.E. (2009). Time-dependent density-functional theory for molecules and molecular solids. J. Mol. Struc. THEOCHEM.

[144] Monti M., Stener M., Coccia E. (2023). Electronic circular dichroism from real-time propagation in state space. J. Chem. Phys.

[145] Biancorosso L., D’Antoni P., Corni S., Stener M., Coccia E. (2024). Time-dependent quantum/continuum modelling of plasmon-enhanced electronic circular dichroism. J. Chem. Phys..

[146] Coccia E., Fregoni J., Guido C.A., Marsili M., Corni S. (2020). Hybrid theoretical models for molecular nanoplasmonics. J. Chem. Phys..

[147] Grobas Illobre P., Marsili M., Corni S., Stener M., Toffoli D., Coccia E. (2021). Time-Resolved Excited-State Analysis of Molecular Electron Dynamics by TDDFT and Bethe–Salpeter Equation Formalisms. J. Chem. Theory Comput..

[148] Scott M., Rehn D.R., Norman P., Dreuw A. (2021). Ab initio excited-state electronic circular dichroism spectra exploiting the third-order algebraic-diagrammatic construction scheme for the polarization propagator. J. Phys. Chem. Lett..

[149] Schirmer J. (1982). Beyond the random-phase approximation: A new approximation scheme for the polarization propagator. Phys. Rev. A.

[150] Fetter A.L., Walecka J.D. (2012). Quantum Theory of Many-Particle Systems.

[151] Dickhoff W.H., Van Neck D.V. (2008). Many-Body Theory Exposed! Propagator Description of Quantum Mechanics in Many-Body Systems.

[152] Dreuw A., Wormit M. (2015). The algebraic diagrammatic construction scheme for the polarization propagator for the calculation of excited states. Wiley Interdiscip. Rev. Comput. Mol. Sci..

[153] Dreuw A., Dempwolff A.L. (2023). Algebraic diagrammatic construction schemes for the simulation of electronic spectroscopies. Theoretical and Computational Photochemistry.

[154] Sokolov A.Y. (2018). Multi-reference algebraic diagrammatic construction theory for excited states: General formulation and first-order implementation. J. Chem. Phys..

[155] Mertins F., Schirmer J. (1996). Algebraic propagator approaches and intermediate-state representations. I. The biorthogonal and unitary coupled-cluster methods. Phys. Rev. A.

[156] Knippenberg S., Rehn D., Wormit M., Starcke J., Rusakova I., Trofimov A., Dreuw A. (2012). Calculations of nonlinear response properties using the intermediate state representation and the algebraic-diagrammatic construction polarization propagator approach: Two-photon absorption spectra. J. Chem. Phys..

[157] Schirmer J. (1991). Closed-form intermediate representations of many-body propagators and resolvent matrices. Phys. Rev. A.

[158] Schirmer J., Trofimov A.B. (2004). Intermediate state representation approach to physical properties of electronically excited molecules. J. Chem. Phys..

[159] Stanton J.F., Bartlett R.J. (1993). The equation of motion coupled-cluster method. A systematic biorthogonal approach to molecular excitation energies, transition probabilities, and excited state properties. J. Chem. Phys..

[160] Krylov A.I. (2008). Equation-of-motion coupled-cluster methods for open-shell and electronically excited species: The hitchhiker’s guide to Fock space. Annu. Rev. Phys. Chem..

[161] Nascimento D.R., DePrince III A.E. (2016). Linear absorption spectra from explicitly time-dependent equation-of-motion coupled-cluster theory. J. Chem. Theory Comput..

[162] Nascimento D.R., DePrince A.E. (2019). A general time-domain formulation of equation-of-motion coupled-cluster theory for linear spectroscopy. J. Chem. Phys..

[163] Friese D.H., Hättig C., Rizzo A. (2016). Origin-independent two-photon circular dichroism calculations in coupled cluster theory. Phys. Chem. Chem. Phys..

[164] Faber R., Ghidinelli S., Hättig C., Coriani S. (2020). Magnetic circular dichroism spectra from resonant and damped coupled cluster response theory. J. Chem. Phys..

[165] Vidal M.L., Feng X., Epifanovsky E., Krylov A.I., Coriani S. (2019). New and efficient equation-of-motion coupled-cluster framework for core-excited and core-ionized states. J. Chem. Theory Comput..

[166] Nanda K.D., Krylov A.I. (2018). The effect of polarizable environment on two-photon absorption cross sections characterized by the equation-of-motion coupled-cluster singles and doubles method combined with the effective fragment potential approach. J. Chem. Phys..

[167] Coriani S., Koch H. (2015). Communication: X-ray absorption spectra and core-ionization potentials within a core-valence separated coupled cluster framework. J. Chem. Phys..

[168] Pulm F., Schramm J., Hormes J., Grimme S., Peyerimhoff S.D. (1997). Theoretical and experimental investigations of the electronic circular dichroism and absorption spectra of bicyclic ketones. Chem. Phys..

[169] Hoerner P., Lee M.K., Schlegel H.B. (2019). Angular dependence of strong field ionization of N_2_ by time-dependent configuration interaction using density functional theory and the Tamm-Dancoff approximation. J. Chem. Phys..

[170] Rüger R., Franchini M., Trnka T., Yakovlev A., van Lenthe E., Philipsen P., van Vuren T., Klumpers B., Soini T. (2022). AMS 2022.1, SCM, Theoretical Chemistry.

